# Accelerating microbial iron cycling promotes re‐cementation of surface crusts in iron ore regions

**DOI:** 10.1111/1751-7915.13646

**Published:** 2020-08-19

**Authors:** Emma J. Gagen, Alan Levett, Anat Paz, Heike Bostelmann, Rafael Borges da Silva Valadares, José Augusto Pires Bitencourt, Markus Gastauer, Gisele Lopes Nunes, Guilherme Oliveira, Paulo M. Vasconcelos, Gene W. Tyson, Gordon Southam

**Affiliations:** ^1^ School of Earth and Environmental Sciences The University of Queensland St Lucia QLD 4072 Australia; ^2^ Australian Centre for Ecogenomics School of Chemistry and Molecular Biosciences The University of Queensland St Lucia QLD 4072 Australia; ^3^ Instituto Tecnológico Vale Belém Pará 66055‐090 Brazil; ^4^ Present address: GFZ German Research Centre for Geosciences Potsdam 14473 Germany; ^5^ Present address: School of Biomedical Sciences Queensland University of Technology Brisbane QLD 4001 Australia

## Abstract

Accelerating microbial iron cycling is an innovative environmentally responsible strategy for mine remediation. In the present study, we extend the application of microbial iron cycling in environmental remediation, to include biocementation for the aggregation and stabilization of mine wastes. Microbial iron reduction was promoted monthly for 10 months in crushed canga (a by‐product from iron ore mining, dominated by crystalline iron oxides) in 1 m^3^ containers. Ferrous iron concentrations reached 445 ppm in treatments and diverse lineages of the candidate phyla radiation dominated pore waters, implicating them in fermentation and/or metal cycling in this system. After a 6‐month evaporation period, iron‐rich cements had formed between grains and 20‐cm aggregates were recoverable from treatments throughout the 1‐m depth profile, while material from untreated and water‐only controls remained unconsolidated. Canga‐adapted plants seeded into one of the treatments germinated and grew well. Therefore, application of this geobiotechnology offers promise for stabilization of mine wastes, as well as re‐formation of surface crusts that underpin unique and threatened plant ecosystems in iron ore regions.

## Graphical abstract

We promoted microbial iron reduction and subsequent iron oxidation, in crushed iron‐rich mine waste at pilot scale (~1 m^3^). Repeated iron cycling led to the formation of new iron oxide cements cementing grains and aggregating the waste material. The technology has promise for accelerating remediation of iron ore mine sites.

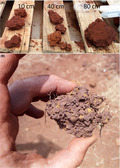

## Introduction

Iron ore deposits that formed from the weathering of banded iron formations in tropical regions are often capped by a hard goethite‐cemented duricrust, known as canga (Dorr, 1964; Shuster *et al*., 2012; Monteiro *et al*., 2014; Gagen *et al*., 2019b). Cangas are extremely resistant to erosion and represent some of the longest‐lived continuously exposed surfaces on Earth (Monteiro *et al*., 2018a). The stability of these kilometre‐scale features is partly the result of repeated biologically mediated dissolution and subsequent reprecipitation of the goethite cements in canga (Monteiro *et al*., 2014; Levett *et al*., 2020b). If the natural geobiological processes that result in cementation and ‘self‐healing’ of canga over geologic time could be accelerated in the present day, stable iron oxide surface crusts may be re‐formed during the rehabilitation of iron ore mines. This represents a significant opportunity for mine site restoration because, in tropical iron ore regions, a unique canga‐associated ecosystem covers the abundant iron reserves demanded by industrial mining activities (Skirycz *et al*., 2014; Gagen *et al*., 2019b).

Under circumneutral conditions, the dissolution of crystalline iron oxides (e.g. goethite, hematite) is the rate‐limiting step in the iron cycling necessary for cementation of canga (Monteiro *et al*., 2014; Levett *et al*., 2020b). We recently enriched a microbial consortium from a canga area, dominated by a novel iron‐reducing *Telmatospirillum* and capable of significant goethite reduction via sugar fermentation (Gagen *et al*., 2019a). Gleaning from those observations, we sought to test at pilot‐scale the potential for fermentation of glucose to drive repeated microbial iron reduction and promote overall re‐cementation of crushed canga from iron ore mining. Our results implicate novel lineages of the candidate phyla radiation in fermentation and/or metal cycling and demonstrate that *in situ* microbial iron cycling leads to the formation of new iron oxide cements that trap smaller fragments at the microscale and aggregate crushed iron ore material at the centimetre scale. This is the first demonstration of this process on a large scale, and we believe that it has significant potential for the stabilization of iron‐oxide‐rich mine waste.

## Results

### Experimental design and considerations

The experimental site was a remote location [6°03′00″S 50°04′50″W], near to the N4 mine in Carajás, Brazil, owned and operated by Vale S.A. Approximately 10 000 kg of crushed iron‐oxide‐rich waste from N4 mine was used in this experiment; therefore, we estimate that each international bulk container (IBC, ~ 1 m^3^) held approximately ~ 2000 kg of rock material. Two experimental controls and two treatments were considered in the experimental design. Untreated canga left exposed in the weather (A) was considered the negative control and provides a glimpse of the natural geobiological processes occurring in crushed canga, in a post‐mining situation. A water‐only control (B) was an experimental control for the effect of adding liquid to crushed canga. Two treatments designed to stimulate microbial iron cycling were as follows: (i) the addition of liquid medium containing glucose and nutrients (C) and (ii) the same medium with microbial inocula added (D), see Figs S1 and S2. These treatments were designed to explore a range of questions that could inform post‐mining application of microbial biotechnologies to accelerate consolidation of crushed canga. Firstly, can indigenous microorganisms in crushed canga drive iron cycling? Does providing a microbial inoculant stimulate iron cycling more than the indigenous microorganisms? Does microbial iron cycling (i.e. ‘treatment’) promote canga aggregation compared to an untreated or watered control? Treatment D was also replicated, and the seeds of local plants provided to the replicate (referred to as treatment E) to determine whether the experimental approach had any adverse effects on the germination or growth of canga plants, which is an important consideration for post‐mining ecosystem restoration attempts.

In keeping with examination of this technology for industry application, a sterilized control was not included, nor was it feasible to sterilize ~ 2000 kg of crushed canga in a remote location of the Carajás Iron Ore Province, Brazil. Apart from a clear plastic cover to reduce exposure to high levels of rainfall (Fig. S1), attempts were not made to control the broader environmental conditions of the experiment, and as such, seed that blew in from the surrounding area and germinated in treatments and controls was allowed to remain, consistent with what might happen in a post‐mining rehabilitation situation (Figs S3 and S4 and File S1).

An overarching aim of the research was to investigate strategies for consolidation and cementation of mine waste; therefore, it was imperative that the canga was not disturbed (i.e. subsampled) throughout the experiment. However, quantitatively assessing consolidation of the crushed material at the end of the experiment proved challenging, as, to the best of our knowledge, an in‐field method for assessing aggregation and cementation of crushed rock material does not currently exist. Use of a soil penetrometer and a range of shear strength apparatus were found to be unsuitable for assessing the strength of the cements between canga clasts, because these methods are highly affected by the presence of the extremely strong and angular canga fragments themselves. We have instead focused on examining the nature and differences of the cements that formed between canga clasts at the end‐point in each experiment, using high‐resolution scanning electron microscopy.

### Pore water chemistry of pilot‐scale experiments

Pore water pH of the two treatments (uninoculated, C and inoculated, D) was between 5.5 and 6.0 throughout the experiment and was not significantly different (*P* < 0.05, Table 1). Pore water ferrous iron in the treatments (C, D) ranged from 290 to 445 ppm (Table 1). After the first week, ferrous iron was not significantly different (*P* > 0.05) between the treatment inoculated with microbial consortia and the uninoculated treatment provided with medium only. The pH of the water‐only control was significantly (*P* < 0.05) lower than treatments (C, D) after the first week (pH 4.5, Table 1) and ferrous iron was never detected in it (significantly different to treatments, *P* < 0.001). Pore waters of the water‐only control were visibly orange, presumably from very fine iron oxides, which often made filtration (0.22 µm) for later geochemical analysis, or collection on Sterivex for DNA analysis, impractical. Soluble Mn was significantly (*P* < 0.05) higher in treatments (inoculated and uninoculated, average across the experiment 9.4–9.8 ppm) than in the water‐only control (average across the experiment = 1.41 ppm, Table S1). Nitrogen oxides were virtually undetected in the uninoculated and inoculated treatments (maximum 0.14 ppm) compared to the water‐only control (12.8–30.2 ppm, Table S2, *P* < 0.001 when considering NOx averaged across the experiment). Acetic and formic acids were the main short chain fatty acids detected in pore waters and they were present in all IBCs, variably throughout the experiment, including the water‐only control (Table S2).

**Table 1:** Pore water ferrous iron and pH in canga consolidation experiment.

	pH			Ferrous iron (ppm)		
	Water‐only control	Uninoculated treatment	Inoculated treatment	Water‐only control	Uninoculated treatment	Inoculated treatment
Wk 1	5.7 ± 0.0^a^	5.7 ± 0.0^b^	5.6 ± 0.1^b^	0.0 ± 0.0^a^	0.0 ± 0.0^b^	22 ± 17^b^
Wk 12	4.7 ± 0.0^a^	5.9 ± 0.1^b^	5.8 ± 0.0^b^	0.0 ± 0.0^a^	308 ± 22^b^	317 ± 17^b^
Wk 24	4.7 ± 0.0^a^	5.5 ± 0.2^b^	5.7 ± 0.1^b^	0.0 ± 0.0^a^	303 ± 26^b^	405 ± 26^b^
Wk 40	4.5 ± 0.1^a^	5.9 ± 0.1^b^	6.0 ± 0.1^b^	0.0 ± 0.0^a^	290 ± 61^b^	445 ± 40^b^

Samples across a row that are followed by different letters are significantly different at *P* < 0.05. Range of detection for the ferrous iron test was 0–1200 ppm.

### Pore water microbial communities

**Fig. 1:**
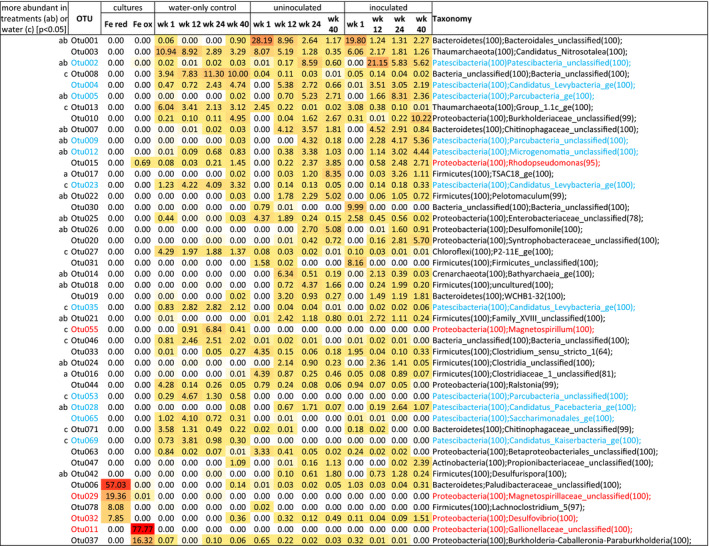
Heatmap denoting abundance of bacterial and archaeal OTUs in each library from triplicate (unless otherwise indicated) pore water samples collected at weeks 1, 12, 24 and 40, and from the iron‐reducing and iron‐oxidizing cultures inoculated to treatment D during construction. Darker colours (red) represent higher abundance while light colours (white) represent lower abundance. The OTUs highlighted in red are from lineages known to contain iron‐cycling organisms. OTUs highlighted in blue classify within the candidate phyla radiation. OTUs with significantly (*P* < 0.05) higher abundance are indicated by lowercase letters in the left column for the uninoculated or inoculated treatment relative to the water‐only control (a, b respectively) and for the water‐only control (c) relative to both treatments. Taxonomy, with confidence shown in brackets, has been shown at phylum level and then only at the highest resolution thereafter, full data is available in File S2. ^a^single sample only ^b^duplicate sample only. In the case of week 1, triplicate samples were pooled prior to DNA extraction.

Pore water microbial communities in the treatments (C, D) were generally dominated by Firmicutes and Bacteroides, which declined through time as Patescibacteria and Proteobacteria came to be the most abundant phyla in weeks 12 to 40 (Fig. S5). In contrast, the water‐only control (B) was dominated by either Proteobacteria or Patescibacteria throughout, while Firmicutes and Bacteroides represented only a maximum of 3.9–5.5% of sequences at any point (Fig. S5). Most major OTUs in both treatments and the water‐only control did not have close (> 95% 16S rRNA gene identity) cultured representatives in the public domain (File S2) highlighting the novelty of the microbial communities in this system. Well‐known iron‐cycling lineages (*Geobacter*, *Shewanella*) were also not abundant in the treatments (Fig. 1). The most abundant OTU in the treatments at week 1 was affiliated with *Bacteroides ovatus* (95.2% 16S rRNA gene identity) and declined through time, as Patescibacteria OTUs increased in abundance in the treatments from week 12 onwards (Fig. 1), coincident with ferrous iron production. By week 24, five of the top ten OTUs in both the treatments were Patescibacteria (OTUs 2, 4, 5, 9, 12) and some of these persisted as abundant OTUs until week 40. A Chitinophagaceae (OTU 7, 94.8% identity across the 16S rRNA gene to *Flavisolibacter rigui*) was a consistently abundant member of the treatments from week 12 to 40 and was never detected above 0.03% in the water‐only control (Fig. 1). *Rhodopseudomonas palustris* (OTU 15) which is known to be capable of light‐dependent iron oxidation (Jiao and Newman, 2007), increased in the treatments through time (comprising 2.3–3.8% of sequences in weeks 24 and 40). In the water‐only control *R. palustris* was notable only at week 40 (1.5% of sequences). By week 40, major OTUs in treatments included those affiliating, albeit distantly at times, with lineages known to be capable of anaerobic fermentation and/or respiration (*Ideonella*, *Methanobacterium, Propionicimonas*, *Pelotomaculum*, *Desulfomonile*, *Desulfovibrio*, *Desulfovirga* spp., Fig. 1 and File S2). The *Ideonella*, *Methanobacterium* and *Propionicimonas* were also abundant in the water‐only control at week 40 (Fig. 1, File S2).

Of the organisms inoculated to treatment D, the *Paludibacter* which dominated the iron‐reducing consortium (OTU 6) was detected at 1.03% at week 1 and 0.3% at week 40 in the inoculated treatment, but was otherwise in low abundance, as in the uninoculated treatment (<0.05% of sequences at all times) and in the water‐only control (0.14% of sequences at week 40 only, Fig. 1). The iron‐reducing *Telmatospirillum* (OTU 29) from the consortium was only detected in low abundance (0.001% of sequences) in week 1 in the inoculated treatment and was detected in the water‐only control at week 24 (0.004% of sequences) The *Desulfovibrio* (OTU 32) in the consortium was detected in the inoculated treatment at all time points (0.04% to 1.5% of sequences), as well as in the uninoculated treatment across weeks 12 to 40 (0.12% to 0.48% of sequences) and in the water‐only control at week 40 (0.36% of sequences, Fig. 1). The *Sideroxydans* OTU dominating the iron‐oxidizing consortium provided to the inoculated treatment at 20 cm during construction was not detected (unsurprisingly in pore waters at 80 cm depth), although the subdominant OTU (*Paraburkholderia*) in that consortium was present in all samples, including the uninoculated treatment and the water‐only control (< 0.001% to 0.65% sequences, Fig. 1). Of lineages detected in the metagenome of the phototrophic consortia (see File S2), only *Rhodopseudomonas* was also an abundant OTU in any pore waters (Fig. 1 and File S2).

The most abundant OTU in the water‐only control at week 1 classified as a *Nitrosotalea* (OTU 3, Fig. 1) as well as in both treatments at time zero. While OTU 3 decreased in relative abundance from week 1 to week 40 in all IBCs, it remained within the top 5 OTUs for the water‐only control at all sampling points. An increasing prevalence of diverse Patescibacteria OTUs over time was also evident in the water‐only control, although for mostly different OTUs than those observed in the treatments. Patescibacteria OTU 23 was present in the water‐only control at week 1 and increased proportionally through the experiment comprising up to 4.2% of sequences at week 12 (never detected above 0.2% in the treatments, Fig. 1). In week 12, four additional Patescibacteria appeared within the top 10 OTUs of the water‐only control (OTUs 53, 65, 69, 35) comprising 2.8–4.7% of the community while only ever detected in treatments at ≤ 0.06% sequences. Patescibacteria OTUs 4, 23 and 35 were major OTUs in the water‐only control in weeks 24 and 40, and of these, OTU 4 was also a major member of the treatments in weeks 12–40 (up to 5.4% of the treatments, Fig. 1). *Magnetospirillum magnetotacticum* was abundant (6.84% of sequences) in the water‐only control in week 12, suggesting that intracellular iron cycling was occurring in this IBC (Faivre *et al*., 2008), potentially linked to nitrite reduction (Li *et al*., 2013) given the abundance of nitrogen oxides present in the water‐only control (Table S2).

**Fig. 2:**
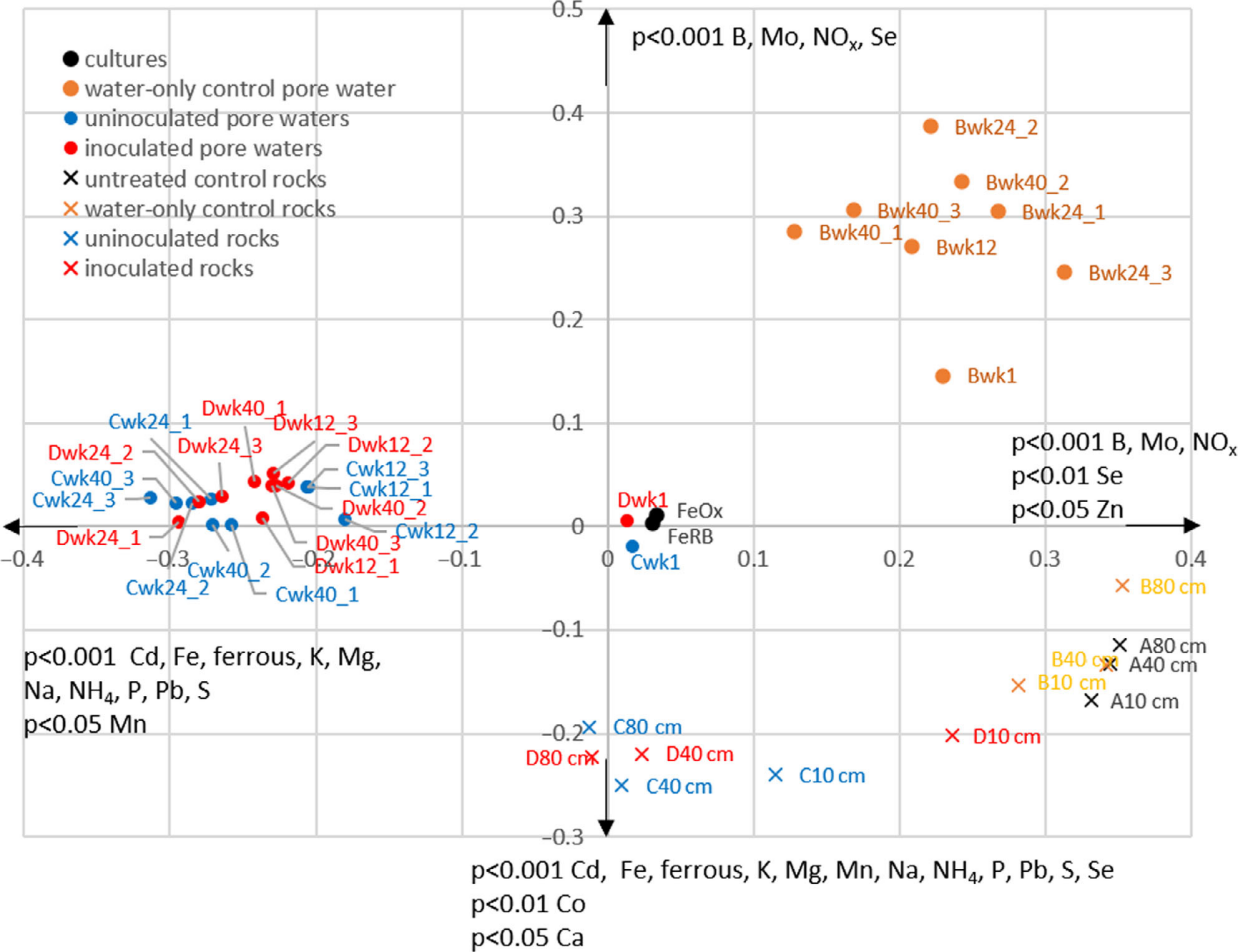
Principle Coordinates Analysis of microbial communities in pore waters and on rocks harvested at end‐point for all samples. The x axis explains 12.6% of the variability in the data, the y axis explains 6.7%. Pore water geochemical data that correlated significantly (Pearson’s correlation coefficient) with direction along the axes are indicated with arrows.

While AMOVA did not indicate any significant differences in the genetic diversity of the microbial community between treatments or the water‐only control, there were OTUs that were significantly (*P* < 0.05) differentially represented in the treatments compared to the water‐only control (indicated in Fig. 1). Furthermore, principal coordinates analysis showed separation of the water‐only control samples at weeks 12, 24 and 40 from the treatments at the same time points, which clustered together (Fig. 2). In that analysis, pore water microbial communities were also distinct from those on rock samples collected at end harvest (week 64; Fig. 2). Results of the microbial community on end harvest rocks are presented in File S1 and Fig. S6.

### Consolidation of canga after drying

**Fig. 3:**
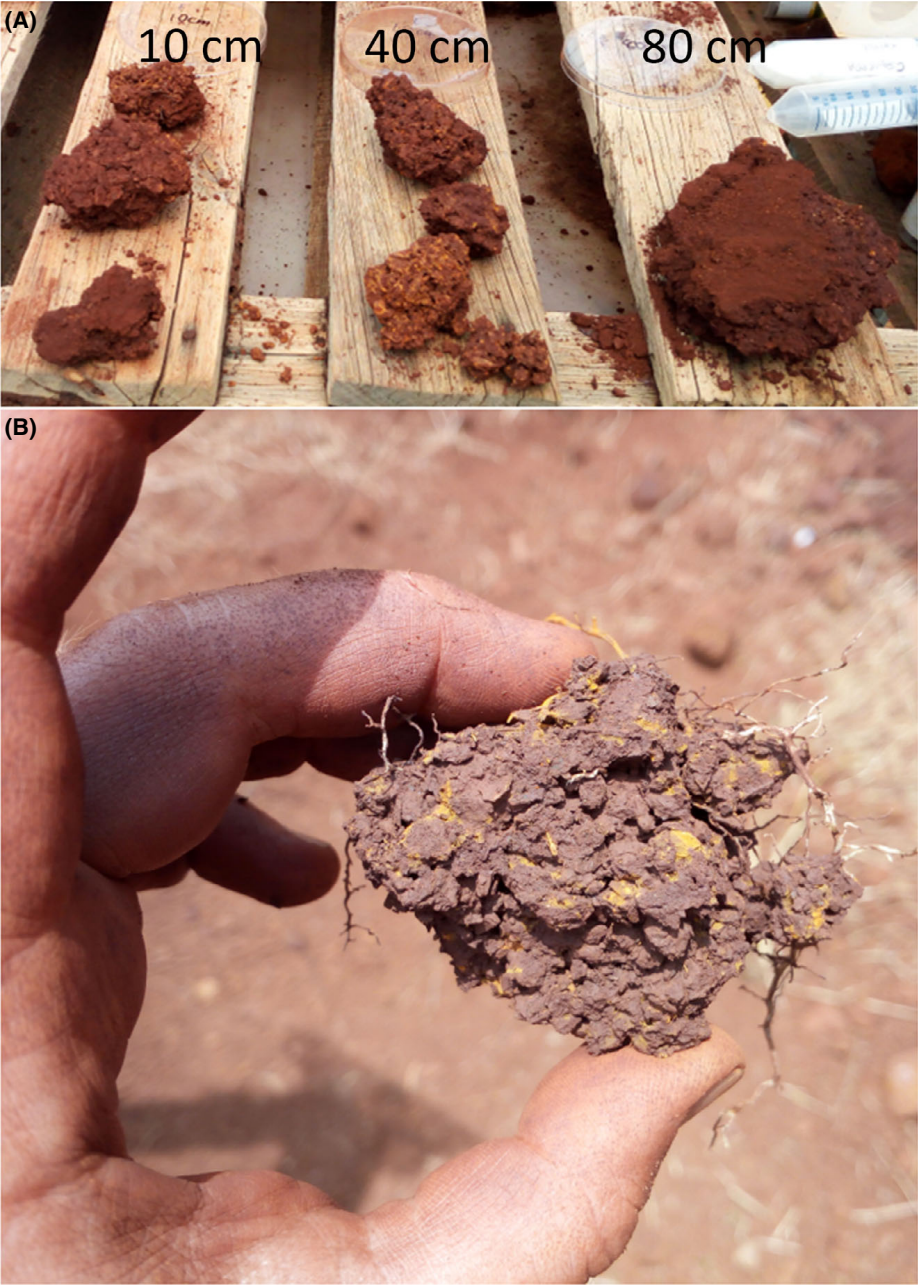
Examples of re‐cemented canga fragments recovered at end harvest from (A) the inoculated treatment (treatment D) at 10 cm, 40 cm, 80 cm; (B) the replicate of this treatment that was also seeded with canga plants (treatment E) at 40 cm depth.

**Fig. 4:**
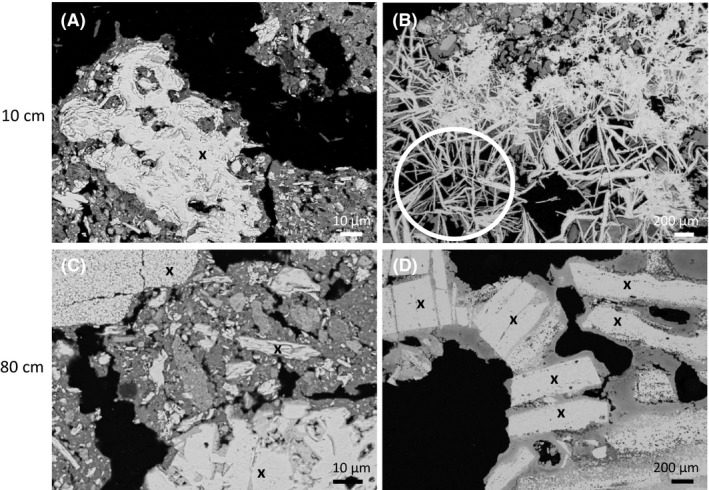
BSE‐SEM micrographs of re‐cemented canga fragments from the inoculated treatment at 10 cm (A, B) and 80 cm (C, D) depth. The higher magnification image (A) is representative of the cementation and infilling of pore spaces that was evident in surface (10cm) samples from the uninoculated treatment and the inoculated treatments (with and without seeding of canga plants) at 10 cm. Cements contain more light elements (see Fig. S7) and therefore appear darker than the materials that they bind (large grain examples of original material are indicated by ‘x’). The occurrence of secondary cements on individual grains (e.g. D) and more commonly the cementation and consolidation of fines by these cements (C) is representative of samples at depth (40–80 cm) from the uninoculated and inoculated treatments. The occurrence of novel, platy iron oxide minerals seen in cross section (circled, B) was only evident in the inoculated treatment at the surface (10 cm) and is a feature that has not been observed in canga samples before. These minerals might be interpreted as platy hematite, formed from the dehydration of ferrihydrite, and partial infilling of cement between the platy minerals is also evident.

**Fig. 5:**
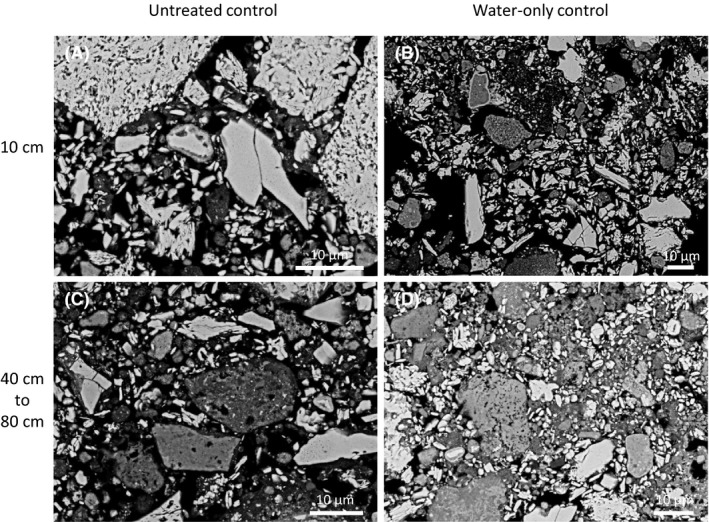
Representative BSE‐SEM micrographs of canga fragments from the untreated control and water‐only control at 10 cm (A, B); and 40 to 80 cm (C, D) depth. In contrast to the treatments (Fig. 4), large grains in the untreated control and the water‐only control are very rarely cemented together (e.g. some cements evident in panel D). Grains in both the untreated and water‐only control appear more densely packed than in the treatments (Fig. 4) due to packing required to embed these extremely crumbly, friable, control samples for polishing.

After a 6‐month dehydration period (i.e. week 64), the IBCs were cut open, and hand samples of consolidated material ~ 5 cm diameter and greater (up to 20 cm diameter) were recoverable from the treatments (e.g. Fig. 3). In contrast, canga in the water‐only control and the untreated control remained unconsolidated (crumbly), except at the edges of the IBCs where the material was weakly held together by fines that had washed down the wall of the IBC. There were no apparent differences in the size of recoverable consolidated material or the level of consolidation between the uninoculated (C) and inoculated (D) treatments. Electron microscopy revealed the heterogeneity of the consolidated material at the microscale and new iron oxide cements, holding together small grains of diverse shapes, and attached to the surface of larger iron oxide grains (Fig. 4). Novel, platy iron oxides (Fig. 4B, plates in cross section appear like needles) that may be secondary hematite were observed in samples at the surface (10 cm) of the inoculated treatment. Samples at depth (40 and 80 cm) demonstrated fine‐grained material trapped in broader scale cements up to 0.5 mm in thickness (e.g. Fig. 4D). The newer cements were more aluminium‐ and phosphorus‐rich than the primary iron oxide grains (Fig. S7). In contrast to treatments, large grains in the untreated control and the water‐only control (Fig. 5) appear aggregated but very rarely cemented together. Samples from both the untreated (A) and water‐only (B) control were crumbly and friable in nature and required packing for embedding.

## Discussion

There is a pressing need for the development of technologies to effectively stabilize rock waste from mining and civil engineering works. Recent advances in biocementation that have focused on microbially induced precipitation of calcite (DeJong *et al*., 2010; Stabnikov *et al*., 2011; McCutcheon *et al*., 2017) offer opportunities for low‐cost stabilization of mine waste and soils. Here, we demonstrate an alternative biocementation approach relevant for iron‐oxide‐rich, low Ca starting materials (e.g. iron ore mine waste) that involves the microbially driven formation of iron oxide cements. In some iron ore regions, natural biogeochemical iron cycling promotes cementation and ‘self‐healing’ of surface duricrusts over geologic time (Monteiro *et al*., 2014; Levett *et al*., 2016; Levett *et al*., 2018; Monteiro *et al*., 2018a; Monteiro *et al*., 2018b). Here, we accelerated the rate‐limiting step in biogeochemical iron cycling in these systems (iron reduction) by providing an easily metabolizable carbon source to promote development of anaerobic conditions and presumably fermentation‐driven dissolution of crystalline iron oxides, based on the work of Gagen and colleagues (2019a). Subsequent re‐oxidation and iron hydrolysis promoted particle aggregation and formation of new iron oxide cements, consolidating the crushed starting material, while control samples remained unconsolidated.

Previously, we demonstrated effective goethite dissolution by a glucose‐fermenting microbial consortium enriched from a canga ecosystem (Gagen *et al*., 2019a). Here, we applied the same approach at large scale (~ 1 m^3^) and found a rapid and effective reduction of iron oxides in canga, sustained over 10 months. Significantly, the provision of glucose and nutrient sources (N, P, K, S) alone was enough to drive effective iron oxide dissolution, even without inoculation of a goethite‐reducing consortium, as iron reduction was not significantly different between the uninoculated and inoculated treatment (Table 1). Members of the inoculated consortia were not abundant in the inoculated treatment, indicating that indigenous microbes are driving iron reduction in this system. However, well‐known iron‐reducing lineages (e.g. *Geobacter*, *Shewanella*) were not abundant in pore waters of the treatments. Rather, pore waters were dominated by uncultivated lineages from the candidate phyla radiation (CPR). Sulphate‐reducing microbial lineages were detected in pore waters intermittently and may have been contributing indirectly to iron reduction by produced bisulphide. Iron‐cycling microbial lineages (e.g. *Geobacter*, *Gallionellaceae*, File S1 and Fig. S6) were more abundant in rock samples collected at end harvest than in pore water samples collected throughout the experiment, suggesting these microorganisms were particle‐associated throughout. This might be expected for direct extracellular electron transfer to a solid surface in the case of *Geobacter* (Kato, 2015) and it seems likely that the iron cycling that was evident in this system was occurring at the microbe–mineral interface.

The remarkable diversity and abundance of microorganisms from lineages without close cultured representatives, warrants further investigation concerning their role in this system. The role of CPR microorganisms in nature remains enigmatic (Castelle *et al*., 2018); however, genomic data indicate that many members are fermenters (Wrighton *et al*., 2014; Danczak *et al*., 2017), which is consistent with their abundance in our treatments fed with glucose and demonstrating anaerobic conditions. In an acetate‐amended aquifer, Wrighton *et al.,* (2014) found proteogenomic evidence to suggest that CPR likely fuel iron reduction and other respiratory metabolisms, via fermentation intermediates. Potentially, the CPR present in the water‐only control plays a role in cycling the nitrogen species detected in that treatment (Castelle *et al*., 2017; Danczak *et al*., 2017). The presence of manganese in pore waters of treatments throughout the experiment also suggests that fermentation‐driven reduction of manganese oxides was occurring (Lovley, 1991).

Iron reduction is the rate‐limiting step in the natural biogeochemical cycling of iron in canga (Levett *et al*., 2020b) and is controlled by the availability of electron donors (carbon or hydrogen) and the presence of anaerobic conditions for microbial respiration to electron acceptors other than oxygen (Gagen *et al*., 2018). Providing an easily metabolizable carbon source facilitated the rapid development of anaerobic conditions in our experiment, confirmed by the presence of ferrous iron in pore waters 6 days after reactor construction [if conditions were oxic, at circumneutral pH ferrous iron would have been rapidly oxidized to ferric iron (Hem and Cropper, 1959)]. Hydrogen generated from the fermentation of glucose and/or its breakdown products was the likely electron donor for direct reduction of the crystalline iron oxides in the system (Wrighton *et al*., 2014; Gagen *et al*., 2019a). Examination of alternative carbon sources (e.g. sucrose, molasses, etc.) would be worthwhile to compare the effectiveness of this approach with economically cheaper inputs to the system.

Anaerobic conditions were maintained at depth throughout the saturated phase of the experiment, based on the high concentrations of ferrous iron detected in pore waters at depth. However, the top 10–20 cm of each treatment was allowed to evaporate naturally each month for the duration of the first phase of the experiment (10 months); thereby oxygenating this portion of the treatment and promoting ferrous iron oxidation and the formation of new iron oxides (see Fig. S2). While we observed that a single dehydration/oxidation event at the end of the experiment (phase 2, drying for 6 months) led to the formation of new iron oxide cements and consolidation of crushed canga at depth (40 and 80 cm, e.g. Fig. 4C and D), the repeated iron‐cycling in the upper 20 cm resulted in more resilient water‐stable aggregates (field observations only, data not shown). Recent work (Levett *et al*., 2020a) has shown that repeated iron cycling can promote iron crust stabilization in a matter of months. Therefore, promoting more rapid iron cycling during drier months of the year would be ideal for uptake of this technology in field conditions.

There may also be a role for the rhizosphere in iron cementation, since plants were present in all IBCs except for the untreated control. The growth of plants in our experiments (Fig. S3), particularly the local canga‐adapted species, is promising and confirms that the intervention we have explored here, does not adversely affect the establishment or re‐establishment of canga plants. Repeated microbial iron reduction and subsequent oxidation offers a useful strategy for *in situ* cementation and/or stabilization of disrupted canga. In practice, this could include using crushed canga stockpiled prior to iron ore mining, for the development of canga refuge areas, where canga‐adapted plants could be maintained. Therefore, there is potential for the approach we have developed to be employed in combination with mine rehabilitation activities where re‐establishment of canga‐adapted plants and canga ecosystems is a priority. We expect that this technology has application for other iron‐oxide‐rich substrates such as mining overburden or waste and that microbially mediated iron cementation represents a possibility for bench or slope stabilization.

## Experimental procedures

### Preparation of microbial consortia

Detailed information on the cultivation, enrichment and identification of organisms in the following iron‐reducing, iron‐oxidizing and phototrophic consortia are available in File S3. Briefly, a goethite‐reducing enrichment culture (Gagen *et al*., 2019a) was grown on anaerobically prepared modified Wolfe’s mineral medium (MWMM; Emerson and Merrill Floyd, 2005) with 30 mM glucose and goethite as iron source. A neutrophilic iron‐oxidizing culture was enriched from a small pool of water perched on canga in Serra Sul, Carajás, Brazil (S06°24′01.7″ W050°22′23.8″) by multiple passages on FeS gradient tubes (with MWMM as the basal medium), as described in Emerson and Merrill Floyd (2005) before upscaling to liquid medium fed daily with FeCl_2_ and air as per Emerson and Merrill Floyd (2005) for use in the field‐scale experiment. A phototrophic consortium established from a pooled mixture of samples collected from Serra Sul and Serra Norte, Carajás, Brazil (small pools perched on canga at each of the following locations, S06°24′01.7″ W050°22′23.8″, S06°23′50.7″ W050°21′19.1″, S06°23′50.5″ W050°21′27.5″, S06°01′37.9″ W050°16′44.9″) was enriched on BG‐11 medium (Stanier *et al*., 1971) on a windowsill in filtered light before being adapted to and grown on MWMM medium for use in the field‐scale experiment. Samples were collected under SISBIO authorization #46320‐1.

### Design of pilot‐scale canga consolidation experiment

Comparative experiments in pilot‐scale canga consolidation tests were: (A) untreated control as canga exposed to the weather; (B) water‐only control; (C) medium‐only (MWMM medium with 30 mM glucose); (D) medium with microbial consortia (the above‐described iron‐reducing consortium added at 60 cm depth, iron‐oxidizing consortium at 20 cm depth and phototrophic consortium at the surface during construction, see Fig. S2); (E) as per D but also surface‐seeded with plants from the region (details below).

### Construction of pilot‐scale canga consolidation experiments

Crushed canga (primarily < 1 cm^3^) from N5W mine, Serra Norte, Carajás, Brazil was provided by Vale S.A., and was shovelled into clean, 1 m^3^ IBCs from which the top had been removed. Generally, liquid (i.e. water or medium, depending on the treatment) was added alternately with loads of canga during construction to allow mixing, to maximize liquid movement into pore spaces throughout each IBC and ensure saturation at the start of the experiment. Based on approximate volumes of liquid that were able to be added to the experiments during construction, the pore space around the canga in each IBCs was estimated to be 15–20% of the volume of the IBC. A PVC pipe (diameter 5 cm), which had incisions placed at ~ 5 cm intervals to allow water to flow into it, was installed in the corner of each IBC during construction. Triplicate pore water samplers (1.5 m long) were inserted into each treatment during construction (Fig. S2). Pore water samplers had been constructed by piercing hard tubing (2.5 mm ID, 4.25 mm OD, 1.5 m long) with a 16 G needle ~ every 3 mm, along 50 cm at one end, and covering this section with 100 µm nylon mesh. This porous end of the samplers was laid horizontally along the canga in the IBCs at approximately 80 cm depth and the remainder of the tubing ran up the side of the IBC, which was connected (silicon sealed) at the top to larger tubing (4.25 mm ID, 6.25 mm OD, 3 cm long) that snugly fit the Luer Lock end of a 50 ml syringe. Due to an oversight during construction, pore water samplers were accidentally not installed in treatment E, and a PVC pipe to determine water depth was not installed in treatment B.

For treatments receiving MWMM medium with glucose, the medium was prepared freshly in 20 l buckets. Where microbial consortia were added (treatments D and E), the iron‐reducing consortium (8 l of well‐grown culture) was added at approximately 60 ‐ 80 cm depth, the iron‐oxidizing consortium (2.5 l) at approximately 20 cm depth and the phototrophic consortium (4 l) on the surface. For treatment E, seeds of *Chloroleucon acaciodes* (Ducke) Barneby & J.W. Grims, *Mimosa pudica* L. and *Mimosa camporum* Benth. (all Fabaceae), provided by Vale S.A., were evenly distributed throughout the surface and buried to ~ 1 cm depth. A clear plastic cover was installed on a sloping angle over the treatments to protect the experiments from rainfall (periodically the cover tore in the wind and was replaced as quickly as practical). Canga in an IBC, untreated and left exposed to the weather served as the experimental control. The chemical composition of the canga used in the experiment was determined by fused disc X‐ray fluorescence (ME‐XRF21n method) at Advanced Laboratory Services, Stafford, Australia and results are presented in Table S3.

### Maintenance and monitoring of pilot‐scale canga consolidation experiments

Once per month, for 10 months, the water level was measured via PVC pipe before IBCs were re‐saturated (B with water (~20 l); treatments C (20–35 l), D (35–38 l) and E (37–47 l) with medium). During the first 12 weeks of the experiment, all IBCs were also re‐saturated with water fortnightly. This watering was to ensure establishment of the plants in treatment E and was done across all IBCs for consistency.

Every three months, pore water was collected from the samplers via 50 ml syringe. The first ~ 70 ml of water in each sampler was discarded to ensure fresh pore water was sampled. Pore water was analysed for pH in the field by MColorpHast™ pH strips 4.0–7.0 (Merck KgaA, Darmstadt, Germany) and for ferrous iron by Chemetrics kit K‐6210D (Watertest Systems, Minto, Australia). Approximately 100 ml pore water was filtered through a Sterivex™ 0.22 µm filter unit (EMD Millipore Corporation, Billerica MA, USA), preserved with 2 ml of Lifeguard® Soil Preservation Solution (Qiagen, Hilden, Germany) and frozen before transport to the laboratory for later DNA extraction. Approximately 15 ml of filtered pore water was collected into autoclaved sealed Hungate tubes with a nitrogen atmosphere before transport to the laboratory for later chemical analysis. For week 1 samples only, pore waters from the triplicate samplers were pooled before geochemical analysis and DNA extraction. All other samplings, the pore waters from triplicates were analysed for each IBC, except where it was not possible to collect pore water from every sampler as was sometimes the case for the water‐only control (B) due to very fine‐grained orange particulate matter clogging the pore water samplers and/or filters.

### Dehydration phase and end harvest of pilot‐scale canga consolidation experiments

After 10 months promoting iron reduction in the treatments (C, D, E), all experiments were allowed to completely dry out by natural evaporation over 6 months (dry season). The front face of the IBCs was then cut open and 20–40 cm of canga from the front face inwards was removed by shovelling, to allow sampling and inspection of the canga in the middle of the experiments. Samples for DNA extraction were collected from ~ 10, 40 and 80 cm depth, into sterile 2 ml tubes and preserved with Lifeguard® Soil Preservation Solution. Hand samples of consolidated canga were transported back to the laboratory in plastic containers.

### DNA extraction, amplicon sequencing, chemical analyses and scanning electron microscopy

Methods pertaining to DNA extraction from pore water and rock samples, 16S rRNA gene amplicon sequencing, sequence processing, chemical analyses of pore waters and microscopy of rock samples at end harvest are available in File S3.

## Conflict of interest

The authors declare no competing financial interests.

## Supporting information


**Fig. S1.** Schematic of field trial design showing untreated control (A), water‐only control (B), uninoculated treatment (C), inoculated treatment (D), a replicate of D that was also seeded with canga‐adapted plants (E) and photograph of the experiment at the field site. Note, in the photograph there are 9 IBCs as this experiment was part of a larger research approach and the additional 4 IBCs are not reported here.

**Fig. S2.** Schematic of experimental design showing crushed canga in an IBC (~1 m^3^). Microbial consortia provided to the inoculated treatment (D) during construction are indicated. IBCs were saturated monthly with either water (B) or medium (C, D). Iron reduction is expected to occur in the treatments (C, D) when saturated, while iron oxidation will occur during the evaporation phases.

**Fig. S3.** Plant cover in the canga reformation experiment weeks 12, 24 and 40.

**Fig. S4.** General overview of plant cover in the experiment at end point (week 64, after 6 months drying) showing plants in those IBCs that had received liquid monthly (panel A includes water‐only control and treatments) compared to the untreated control (panel B) which was left untreated and exposed to natural conditions.

**Fig. S5.** Proportion of bacterial and archaeal sequence by phylum present in porewaters of the water‐only control (B), uninoculated (C) and inoculated (D) treatments, throughout the experiment (weeks 1, 12, 24 and 40, left panel) and in end‐harvest (week 64) rocks in the untreated control (A), water‐only control (B), uninoculated (C) and inoculated (D) treatments 10 cm, 40 cm and 80 cm from the surface (right panel). Porewater data are the average of triplicate samples except for week 1 where triplicates were pooled before DNA extraction and for the water‐only control week 12 where only a single sample was obtained.

**Fig. S6.** Heatmap of major OTUs from most abundant (red) to least abundant (white) shown as a proportion (%) of sequences in each library, from rock samples collected from the top (10 cm), middle (40 cm) and bottom (80 cm) of each IBC at end harvest (64 weeks). OTUs highlighted in red are from lineages known to contain iron cycling organisms. The OTU highlighted in blue classifies within the candidate phyla radiation. Taxonomy has been shown at phylum level and then only at the highest resolution thereafter, full data is available in File S2.

**Fig. S7.** Representative EDS spectra of iron oxide grains (B, D) and the new iron oxides (C) that formed around them, cementing smaller fragments. New cements contain more aluminium and phosphorus than original grains.

**Table S1.** Trace metals in pore waters from canga reformation experiment (ppm).

**Table S2.** Glucose, short chain fatty acids, nitrogen oxides, ammonium and phosphates (ppm) detected in pore waters from canga reformation experiment weeks 12, 24 and 40.

**Table S3.** Composition (%) of canga used in the experiment, determined by fused disc X‐ray fluorescence.

**File S1.** Supplementary Results.

**File S2.** Sequence data and analysis files for amplicon data clustered into Operational Taxonomic Units at ≤ 0.03 distance (first seven sheets) and metagenomic analysis of phototrophic consortium (eighth sheet).

**File S3.** Supplementary Methods.
