# Supplementary material for: Accelerating microbial iron cycling promotes re‐cementation of surface crusts in iron ore regions
Source: Microb Biotechnol. 2020 Aug 19;13(6):1960–71. doi: 10.1111/1751-7915.13646 (PMC7533318; doi:10.1111/1751-7915.13646)
Supplement: Supplementary file 1 — Fig. S1. Schematic of field trial design showing untreated control (A), water‐only control (B), uninoculated treatment (C), inoculated treatment (D), a replicate of D that was also seeded with canga‐adapted plants (E) and photograph of the experiment at the field site. Note, in the photograph there are 9 IBCs as this experiment was part of a larger research approach and the additional 4 IBCs are not reported here. [file MBT2-13-1960-s001.pdf]

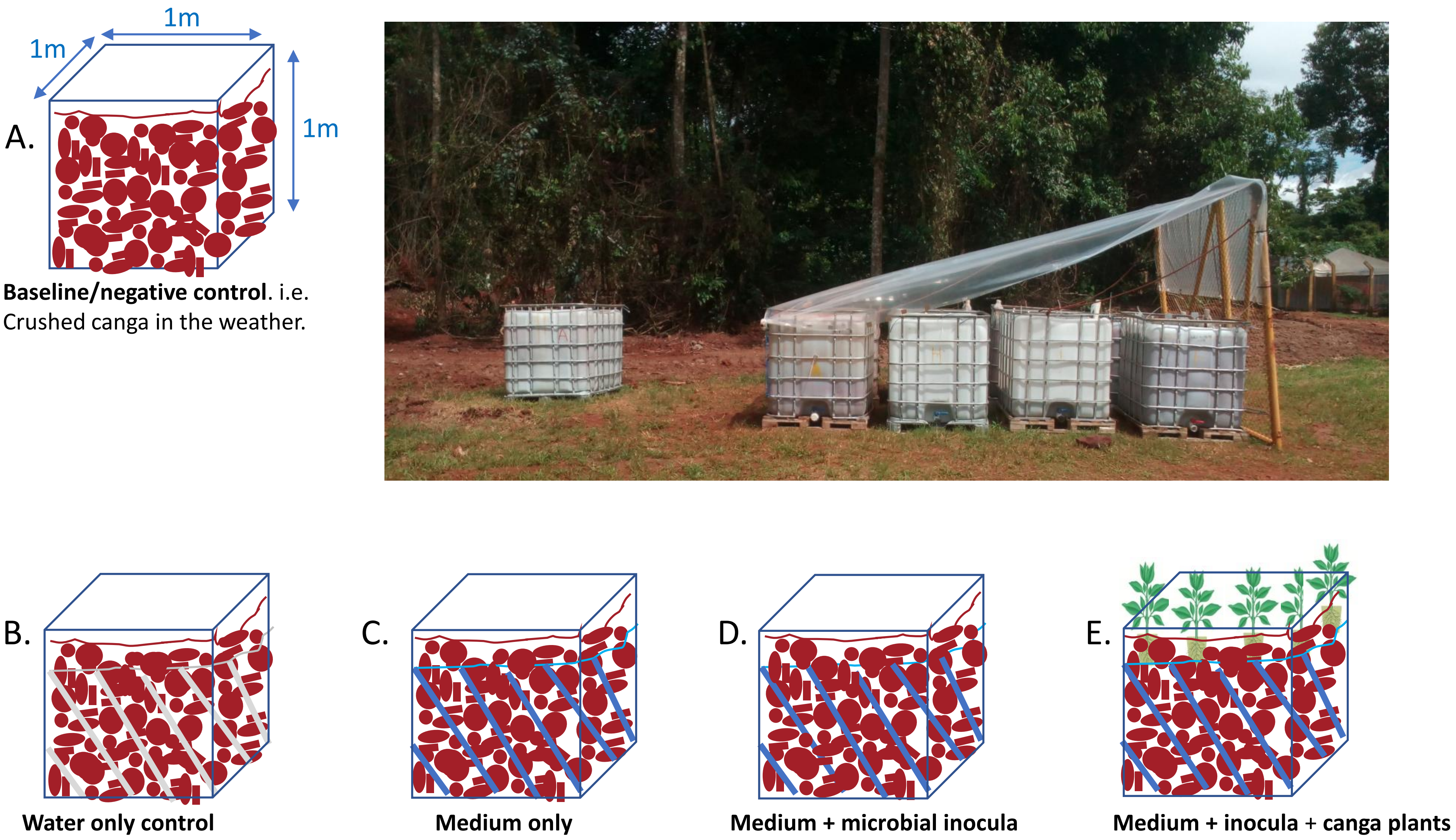

Figure S1. Schematic of field trial design showing untreated control (A), water-only control (B), uninoculated treatment (C), inoculated treatment (D), a replicate of D that was also seeded with canga-adapted plants (E) and photograph of the experiment at the field site. Note, in the photograph there are 9 IBCs as this experiment was part of a larger research approach and the additional 4 IBCs are not reported here.
