# Supplementary material for: Accelerating microbial iron cycling promotes re‐cementation of surface crusts in iron ore regions
Source: Microb Biotechnol. 2020 Aug 19;13(6):1960–71. doi: 10.1111/1751-7915.13646 (PMC7533318; doi:10.1111/1751-7915.13646)
Supplement: Supplementary file 2 — Fig. S2. Schematic of experimental design showing crushed canga in an IBC (~1 m3). Microbial consortia provided to the inoculated treatment (D) during construction are indicated. IBCs were saturated monthly with either water (B) or medium (C, D). Iron reduction is expected to occur in the treatments (C, D) when saturated, while iron oxidation will occur during the evaporation phases. [file MBT2-13-1960-s002.pdf]

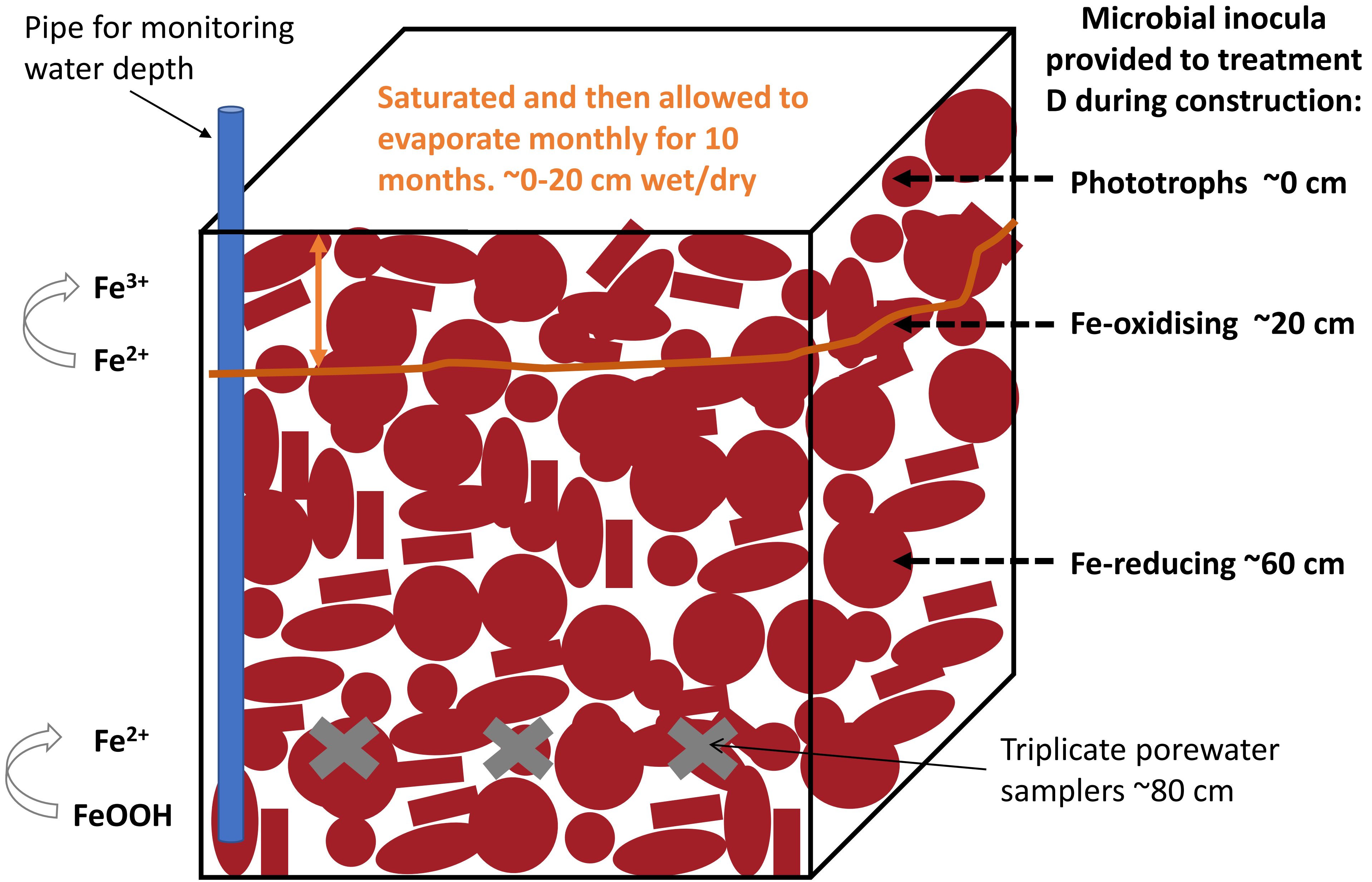

Figure S2. Schematic of experimental design showing crushed canga in an IBC (~1m^3). Microbial consortia provided to the inoculated treatment (D) during construction are indicated. IBCs were saturated monthly with either water (B) or medium (C, D). Iron reduction is expected to occur in the treatments (C, D) when saturated, while iron oxidation will occur during the evaporation phases.
