# Supplementary figures and images for: Accelerating microbial iron cycling promotes re‐cementation of surface crusts in iron ore regions
Source: Microb Biotechnol. 2020 Aug 19;13(6):1960–71. doi: 10.1111/1751-7915.13646 (PMC7533318; doi:10.1111/1751-7915.13646)

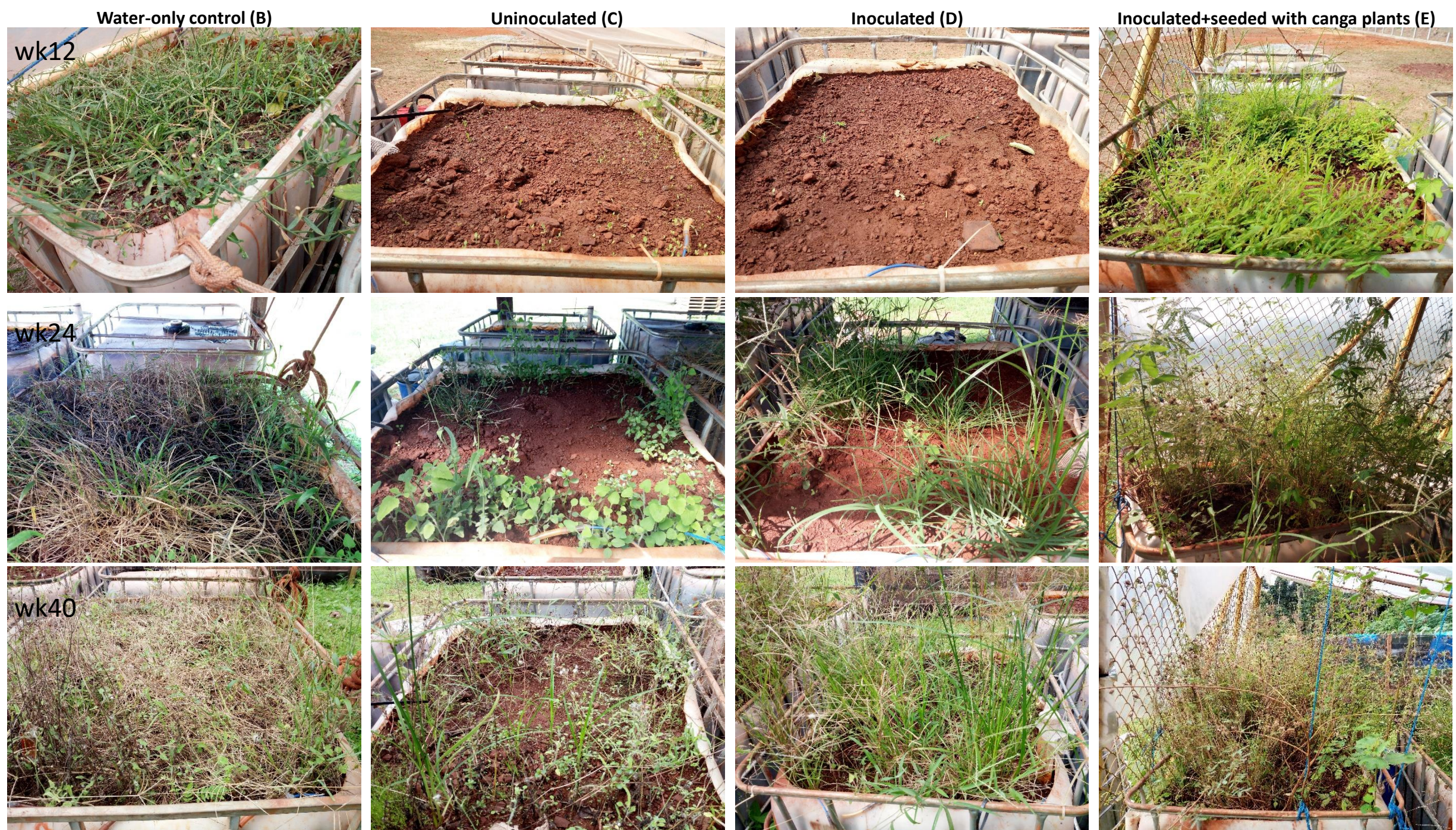

**Figure S3.** Plant cover in the canga reformation experiment weeks 12, 24 and 40

Supplement: Supplementary file 3 — Fig. S3. Plant cover in the canga reformation experiment weeks 12, 24 and 40. [file MBT2-13-1960-s003.pdf]
