# Supplementary material for: Accelerating microbial iron cycling promotes re‐cementation of surface crusts in iron ore regions
Source: Microb Biotechnol. 2020 Aug 19;13(6):1960–71. doi: 10.1111/1751-7915.13646 (PMC7533318; doi:10.1111/1751-7915.13646)
Supplement: Supplementary file 4 — Fig. S4. General overview of plant cover in the experiment at end point (week 64, after 6 months drying) showing plants in those IBCs that had received liquid monthly (panel A includes water‐only control and treatments) compared to the untreated control (panel B) which was left untreated and exposed to natural conditions. [file MBT2-13-1960-s004.pdf]

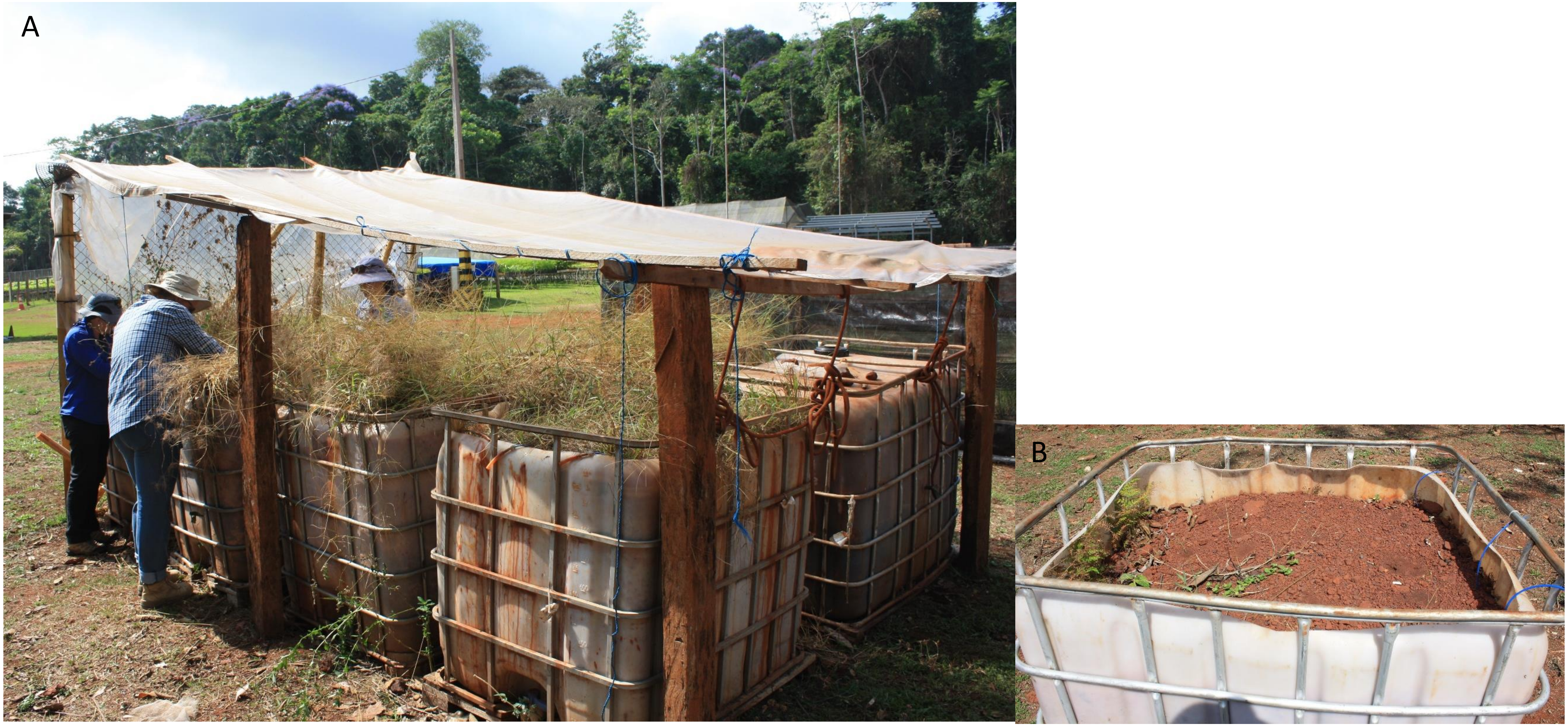

Figure S4. General overview of plant cover in the experiment at end point (week 64, after 6 months drying) showing plants in those IBCs that had received liquid monthly (panel A includes water-only control and treatments) compared to the untreated control (panel B) which was left untreated and exposed to natural conditions.
