# Supplementary material for: Accelerating microbial iron cycling promotes re‐cementation of surface crusts in iron ore regions
Source: Microb Biotechnol. 2020 Aug 19;13(6):1960–71. doi: 10.1111/1751-7915.13646 (PMC7533318; doi:10.1111/1751-7915.13646)
Supplement: Supplementary file 5 — Fig. S5. Proportion of bacterial and archaeal sequence by phylum present in porewaters of the water‐only control (B), uninoculated (C) and inoculated (D) treatments, throughout the experiment (weeks 1, 12, 24 and 40, left panel) and in end‐harvest (week 64) rocks in the untreated control (A), water‐only control (B), uninoculated (C) and inoculated (D) treatments 10 cm, 40 cm and 80 cm from the surface (right panel). Porewater data are the average of triplicate samples except for week 1 where triplicates were pooled before DNA extraction and for the water‐only control week 12 where only a single sample was obtained. [file MBT2-13-1960-s005.pdf]

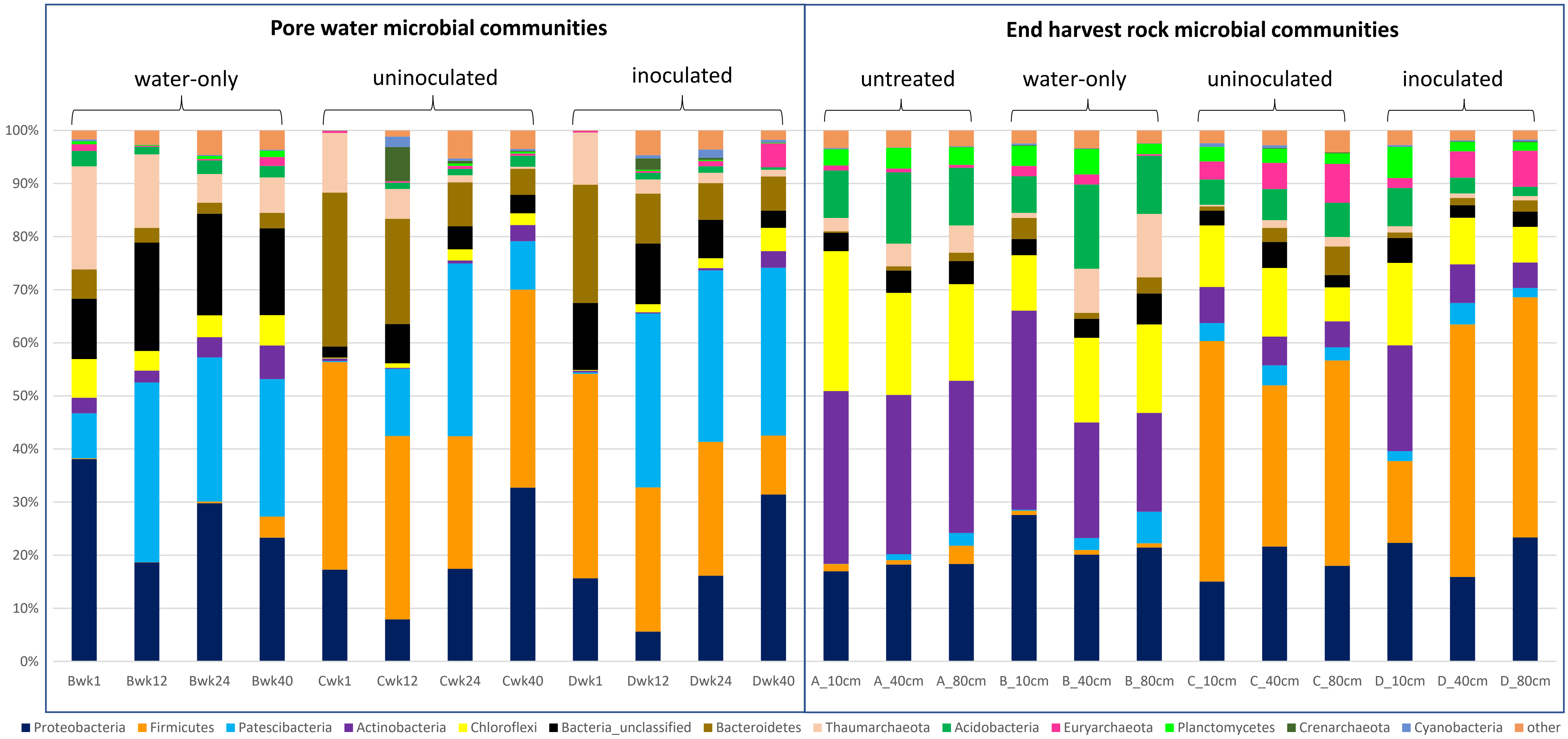

Figure S5. Proportion of bacterial and archaeal sequence by phylum present in porewaters of the water-only control (B), uninoculated (C) and inoculated (D) treatments, throughout the experiment (weeks 1, 12, 24 and 40, left panel) and in end-harvest (week 64) rocks in the untreated control (A), water-only control (B), uninoculated (C) and inoculated (D) treatments 10cm, 40cm and 80cm from the surface (right panel). Porewater data are the average of triplicate samples except for week 1 where triplicates were pooled before DNA extraction and for the water-only control week 12 where only a single sample was obtained.
