# Supplementary material for: Accelerating microbial iron cycling promotes re‐cementation of surface crusts in iron ore regions
Source: Microb Biotechnol. 2020 Aug 19;13(6):1960–71. doi: 10.1111/1751-7915.13646 (PMC7533318; doi:10.1111/1751-7915.13646)
Supplement: Supplementary file 6 — Fig. S6. Heatmap of major OTUs from most abundant (red) to least abundant (white) shown as a proportion (%) of sequences in each library, from rock samples collected from the top (10 cm), middle (40 cm) and bottom (80 cm) of each IBC at end harvest (64 weeks). OTUs highlighted in red are from lineages known to contain iron cycling organisms. The OTU highlighted in blue classifies within the candidate phyla radiation. Taxonomy has been shown at phylum level and then only at the highest resolution thereafter, full data is available in File S2. [file MBT2-13-1960-s006.pdf]

	untreated control			water-only control			uninoculated			inoculated			
OTU	10cm	40cm	80cm	10cm	40cm	80cm	10cm	40cm	80cm	10cm	40cm	80cm	Taxonomy
Otu016	0.00	0.00	0.02	0.00	0.04	0.00	9.91	5.40	14.63	1.81	6.49	23.43	Firmicutes(100);Clostridiaceae_1_unclassified(81);
Otu048	0.00	0.00	0.00	0.00	0.02	0.00	4.65	12.80	9.02	0.43	18.54	5.75	Firmicutes(100);Clostridiaceae_1_unclassified(100);
Otu027	4.99	2.97	3.82	0.63	1.30	2.19	0.94	0.17	0.12	5.96	0.32	0.48	Chloroflexi(100);P2-11E_ge(100);
Otu111	0.00	0.00	0.01	0.09	0.00	0.00	18.54	0.67	0.00	3.39	0.02	0.02	Firmicutes(100);Clostridium_sensu_stricto_6(86);
Otu114	5.26	4.94	5.18	1.41	2.89	2.20	0.02	0.09	0.02	0.31	0.08	0.02	Actinobacteria(100);Conexibacter(86);
Otu058	0.72	0.83	0.56	0.42	0.79	1.16	4.56	3.52	0.93	1.25	1.84	1.02	Chloroflexi(100);JG30-KF-AS9_ge(100);
Otu068	0.02	0.00	0.02	0.00	0.02	0.00	0.75	3.18	1.65	0.96	3.93	6.65	Euryarchaeota(100);Thermoplasmata_unclassified(100);
Otu104	3.62	1.65	1.53	4.74	0.90	0.29	0.24	0.02	0.00	3.43	0.02	0.02	Actinobacteria(100);Sinomonas(100);
Otu128	2.29	2.50	2.01	1.41	3.10	1.92	0.39	0.19	0.31	1.46	0.50	0.14	Proteobacteria(100);uncultured(95);
Otu153	3.59	3.92	3.28	0.85	2.44	1.63	0.00	0.03	0.06	0.22	0.06	0.00	Actinobacteria(100);Acidothermus(100);
Otu003	0.09	1.68	1.47	0.16	2.23	5.39	0.10	1.35	1.61	0.56	0.68	0.72	Thaumarchaeota(100);Candidatus_Nitrosotalea(100);
Otu148	0.47	3.71	2.10	1.10	4.39	2.21	0.10	0.00	0.00	0.65	0.00	0.04	Acidobacteria(100);Subgroup_2_ge(100);
Otu013	0.00	1.04	2.18	0.07	5.07	5.18	0.00	0.00	0.00	0.06	0.06	0.00	Thaumarchaeota(100);Group_1.1c_ge(100);
Otu077	0.00	0.00	0.00	0.00	0.00	0.00	0.00	2.40	3.30	0.01	2.04	5.51	Proteobacteria(100);Gallionellaceae_unclassified(100);
Otu138	0.00	0.00	0.00	0.00	0.00	0.00	0.00	1.90	4.28	0.00	6.35	0.24	Firmicutes(100);Firmicutes_unclassified(100);
Otu088	0.09	0.04	0.01	0.75	0.66	0.03	2.34	1.71	5.64	0.54	0.02	0.02	Euryarchaeota(100);Marine_Group_II_ge(100);
Otu097	1.10	1.44	1.32	0.38	1.26	1.63	0.70	0.86	0.31	0.92	0.84	0.58	Chloroflexi(100);Chloroflexi_unclassified(100);
Otu004	0.00	0.00	0.12	0.00	0.43	1.25	0.34	2.43	0.93	0.49	2.37	0.78	Patescibacteria(100);Candidatus_Levybacteria_ge(100);
Otu209	0.00	0.00	0.00	1.22	0.08	0.07	0.29	0.85	0.08	3.55	1.50	0.48	Actinobacteria(100);Actinokineospora(51);
Otu199	1.30	1.68	2.28	0.28	1.43	0.78	0.00	0.05	0.02	0.09	0.04	0.02	Actinobacteria(100);IMCC26256_ge(100);
Otu165	0.54	0.28	0.42	0.54	0.53	0.37	1.59	0.03	0.00	2.61	0.30	0.14	Actinobacteria(100);Sinomonas(100);
Otu205	0.00	0.00	0.00	0.00	0.00	0.00	0.77	0.03	0.00	6.16	0.00	0.00	Firmicutes(100);Paenibacillus(97);
Otu220	0.92	0.98	0.81	0.59	0.90	0.83	0.29	0.09	0.04	0.56	0.22	0.08	Chloroflexi(100);Chloroflexi_unclassified(100);
Otu294	1.03	1.63	1.49	0.26	0.83	0.40	0.05	0.03	0.06	0.19	0.08	0.00	Actinobacteria(100);Conexibacter(91);
Otu266	0.54	1.25	1.00	1.20	0.53	0.70	0.27	0.07	0.04	0.05	0.06	0.10	Proteobacteria(100);Acidibacter(100);
Otu130	0.00	0.00	0.00	0.00	0.00	0.00	1.52	2.71	0.33	0.06	0.00	1.04	Proteobacteria(100);Geobacter(100);
Otu207	0.52	0.21	0.43	0.31	0.34	0.44	0.05	0.76	1.38	0.04	0.44	0.62	Actinobacteria(100);Solirubrobacteraceae_unclassified(97);
Otu007	0.00	0.00	0.00	0.00	0.00	0.00	0.05	2.04	2.49	0.01	0.44	0.42	Bacteroidetes(100);Chitinophagaceae_unclassified(100);
Otu186	0.74	0.68	0.48	0.52	0.71	0.98	0.31	0.09	0.17	0.43	0.18	0.04	Proteobacteria(100);Rhodoplanes(60);
Otu064	0.00	0.00	0.00	0.00	0.00	0.00	4.19	0.22	0.00	0.86	0.02	0.02	Firmicutes(100);Clostridium_sensu_stricto_6(100);
Otu231	0.09	0.91	0.60	0.52	0.86	0.20	0.31	0.02	0.00	1.69	0.02	0.08	Acidobacteria(100);Subgroup_2_ge(100);
Otu015	1.44	0.19	0.18	0.73	0.15	0.54	0.63	0.05	0.10	1.07	0.10	0.02	Proteobacteria(100);Rhodopseudomonas(95);
Otu224	0.47	0.42	0.48	0.47	1.07	1.16	0.00	0.00	0.00	0.91	0.00	0.00	Proteobacteria(100);MND1(100);
Otu156	0.16	0.32	0.19	0.23	0.32	0.33	0.63	0.66	0.27	0.92	0.62	0.30	Proteobacteria(100);Reyranella(100);
Otu052	0.00	0.00	0.00	0.00	0.00	0.00	0.05	0.38	0.89	0.30	0.14	3.15	Firmicutes(100);Clostridium_sensu_stricto_12(100);
Otu121	0.47	0.30	0.37	0.42	0.23	0.29	0.29	0.74	0.31	0.46	0.66	0.24	Proteobacteria(100);Reyranella(100);
Otu166	0.18	0.27	0.28	0.28	0.68	0.53	0.14	0.41	1.07	0.37	0.18	0.24	Proteobacteria(100);Xanthobacteraceae_unclassified(87);
Otu143	0.00	0.00	0.00	0.00	0.00	0.03	0.02	1.54	1.77	0.02	0.74	0.40	Proteobacteria(100);Curvibacter(80);
Otu124	0.02	0.44	0.68	0.23	0.79	1.42	0.31	0.07	0.00	0.33	0.02	0.14	Proteobacteria(100);Rhodanobacteraceae_unclassified(99);

Figure S6. Heatmap of major OTUs from most abundant (red) to least abundant (white) shown as a proportion (%) of sequences in each library, from rock samples collected from the top (10 cm), middle (40 cm) and bottom (80 cm) of each IBC at end harvest (64 weeks). OTUs highlighted in red are from lineages known to contain iron cycling organisms. The OTU highlighted in blue classifies within the candidate phyla radiation. Taxonomy has been shown at phylum level and then only at the highest resolution thereafter, full data is available in File S2.
