# Supplementary material for: Accelerating microbial iron cycling promotes re‐cementation of surface crusts in iron ore regions
Source: Microb Biotechnol. 2020 Aug 19;13(6):1960–71. doi: 10.1111/1751-7915.13646 (PMC7533318; doi:10.1111/1751-7915.13646)
Supplement: Supplementary file 7 — Fig. S7. Representative EDS spectra of iron oxide grains (B, D) and the new iron oxides (C) that formed around them, cementing smaller fragments. New cements contain more aluminium and phosphorus than original grains. [file MBT2-13-1960-s007.pdf]

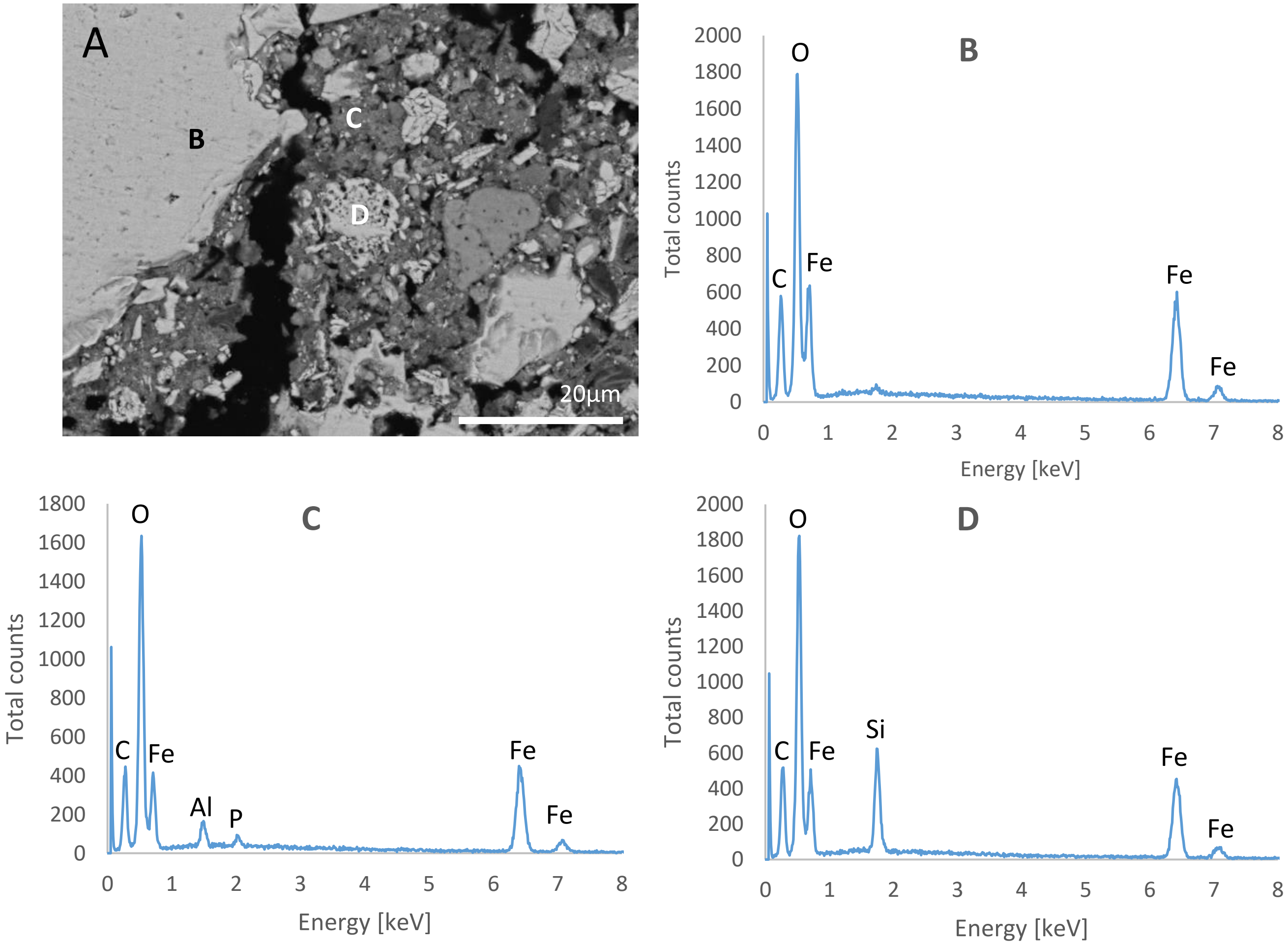

Figure S7. Representative EDS spectra of iron oxide grains (B, D) and the new iron oxides (C) that formed around them, cementing smaller fragments. New cements contain more aluminium and phosphorus than original grains.
