# Supplementary material for: Accelerating microbial iron cycling promotes re‐cementation of surface crusts in iron ore regions
Source: Microb Biotechnol. 2020 Aug 19;13(6):1960–71. doi: 10.1111/1751-7915.13646 (PMC7533318; doi:10.1111/1751-7915.13646)
Supplement: Supplementary file 8 — Table S1. Trace metals in pore waters from canga reformation experiment (ppm). [file MBT2-13-1960-s008.pdf]

Supporting Information Table S1.

Table S1. Trace metals in pore waters from canga reformation experiment (ppm)

Week and treatment or control		**Al**	**Ca**	**Fe**	**K**	**Mg**	**Mn**	**Na**	**P**	**S**	**Zn**
1	Water-only control [a]	0.04	9.24	0.08	8.04	4.80	1.27	18.57	0.00	0.24	0.09
1	Uninoculated[a]	0.10	20.77	155.28	17.44	15.68	16.81	164.39	0.14	10.48	0.30
1	Inoculated [a]	0.12	13.74	113.50	13.87	12.78	15.54	144.22	0.00	10.17	0.45
12	Water-only control [a]	0.01	8.75	0.12	4.98	5.42	2.24	15.75	0.00	0.22	0.10
12	Uninoculated	0.00± 0.00[c]	6.23± 0.30[c]	316.47± 22.98[c]	14.98± 0.13[c]	11.94± 0.59[c]	9.03± 0.29[c]	159.76± 2.55[c]	0.31± 0.03[c]	20.40± 10.53[c]	0.07± 0.00[c]
12	Inoculated	0.01± 0.01[c]	11.24± 1.17[d]	244.98± 30.93[c]	15.35± 0.51[c]	11.13± 0.75[c]	7.75± 0.93[c]	122.83± 1.63[d]	0.25± 0.05[c]	37.96± 3.84[c]	0.08± 0.02[c]
24	Water-only control [b]	0.00± 0.00	6.19± 0.40	0.05± 0.03	3.45± 0.83	3.59± 0.37	1.30± 0.32	14.73± 0.07	0.00± 0.00	0.64± 0.15	0.23± 0.08
24	Inoculated [b]	0.03± 0.03	9.61± 0.90	240.56± 3.78	15.35± 0.23	10.78± 0.08	8.25± 0.75	137.82± 5.72	0.18± 0.01	43.32± 6.76	0.07± 0.02
40	Water-only control	0.01± 0.00[e]	3.48± 0.20[e]	0.00± 0.00[e]	2.42± 0.37[e]	2.20± 0.12[e]	0.83± 0.14[e]	13.05± 0.26[e]	0.01± 0.01[e]	0.66± 0.16[e]	0.22± 0.03[e]
40	Uninoculated	0.03± 0.01[e]	4.03± 0.41[e]	176.84± 12.91[f]	15.48± 0.15[f]	9.14± 0.24[f]	3.49± 0.64[f]	176.94± 1.24[f]	0.20± 0.02[f]	17.76± 6.37[e]	0.11± 0.01[f]
40	Inoculated	0.01± 0.01[e]	8.68± 0.70[f]	285.05± 14.93[g]	12.36± 0.40[g]	10.51± 0.26[g]	6.21± 0.47[g]	123.02± 2.38[g]	0.31± 0.03[g]	20.37± 6.79[e]	0.14± 0.04[ef]

[a] Single sample only. In the case of week 1, triplicates were pooled before analysis; [b] duplicate samples only due to sample loss during transit. All week 24 samples for ‘Uninoculated’ smashed during transit and were unable to be analysed. Data are the average of triplicates unless otherwise indicated, and the standard error of the mean is given. Samples were filtered (0.22 µm) in the field before transport to the laboratory, therefore any metals adsorbed to particles (i.e. < 100 µm to pass through mesh surrounding the pore water samplers, but > 0.22 µm fraction) will not have been recovered in this analysis. This is evident in pore water Fe concentrations appearing lower here than Fe^{2+} measured on unfiltered pore waters (Table 1). Samples collected at the same time point for which trace metal concentrations are significantly different ($p<0.05$) are indicated by superscript letters (c and d for week 12; e, f and g for week 40).
