# Supplementary material for: Accelerating microbial iron cycling promotes re‐cementation of surface crusts in iron ore regions
Source: Microb Biotechnol. 2020 Aug 19;13(6):1960–71. doi: 10.1111/1751-7915.13646 (PMC7533318; doi:10.1111/1751-7915.13646)
Supplement: Supplementary file 9 — Table S2. Glucose, short chain fatty acids, nitrogen oxides, ammonium and phosphates (ppm) detected in pore waters from canga reformation experiment weeks 12, 24 and 40. [file MBT2-13-1960-s009.pdf]

Supporting Information Table S2.

Table S2. Glucose, short chain fatty acids, nitrogen oxides, ammonium and phosphates (ppm) detected in pore waters from canga reformation experiment weeks 12, 24 and 40

Week and treatment or control		**Glucose**	**Succinic**	**Lactic**	**Formic**	**Acetic**	**NOx**	**NH_4**	**PO_4**
12	Water-only control [a]	bdl	bdl	bdl	6.11	0.23	30.20	1.04	0.10
12	Uninoculated	0.78± 0.78	bdl	bdl	8.20± 0.23[c]	1.48± 0.22[c]	0.07± 0.07[c]	3.66± 0.17[c]	0.07± 0.02[c]
12	Inoculated	bdl	bdl	bdl	6.51± 0.50[d]	0.42± 0.22[d]	0.14± 0.07[c]	8.15± 0.29[d]	0.06± 0.01[c]
24	Water-only control [b]	nd	bdl	bdl	1.14± 0.18	0.73± 0.17	19.68± 1.38	0.85± 0.45	0.02± 0.01
24	Inoculated [b]	nd	bdl	bdl	0.70± 0.02	0.70± 0.06	0.02± 0.02	13.60± 0.90	0.00± 0.00
40	Water-only control	bdl	0.15± 0.15	0.13± 0.13[e]	0.62± 0.32[e]	1.11± 0.03[e]	12.87± 0.42[e]	0.89± 0.24[e]	0.13± 0.02[e]
40	Uninoculated	0.06± 0.06	bdl	bdl	0.33± 0.17[e]	0.63± 0.36[f]	0.02± 0.02[f]	10.55± 0.62[f]	0.05± 0.03[f]
40	Inoculated	bdl	bdl	0.16± 0.08[e]	0.57± 0.05[e]	0.77± 0.44[ef]	0.01± 0.01[f]	5.22± 1.38[g]	0.08± 0.05[ef]

[a] single sample only; [b] duplicate sample only; nd: no data; bdl: below detection limit. Data are the average of triplicate samples unless otherwise indicated and the standard error of the mean is given. Week 24 samples for the uninoculated treatment smashed during transit and were unable to be analysed. Propionic, isobutyric, butyric, valeric and isovaleric acids and nitrite, were below detection limit in all samples. Samples collected at the same time point that are significantly different to each other ($p<0.05$) are indicated by superscript letters (c and d for week 12; e, f and g for week 40).
