# Supplementary material for: Accelerating microbial iron cycling promotes re‐cementation of surface crusts in iron ore regions
Source: Microb Biotechnol. 2020 Aug 19;13(6):1960–71. doi: 10.1111/1751-7915.13646 (PMC7533318; doi:10.1111/1751-7915.13646)
Supplement: Supplementary file 10 — Table S3. Composition (%) of canga used in the experiment, determined by fused disc X‐ray fluorescence. [file MBT2-13-1960-s010.pdf]

Supporting Information Table S3.

Table S3. Composition (%) of canga used in the experiment, determined by fused disc X-ray fluorescence

Al_2O_3	Ba	CaO	Cl	Cr_2O_3	CuO	Fe_2O_3	K_2O	MgO	Mn	Na_2O	P_2O_5	Pb	S	SiO_2	Sn	Sr	TiO_2	V	Zn	Zr	LOI	Total
1.42	7e-3	0.02	4e-3	2e-3	7.5e-3	93.66	0.02	0.02	0.06	8e-3	0.42	4e-3	0.01	1.54	1e-3	3e-3	0.27	0.01	2e-3	0.01	2.45	99.96

LOI: loss on ignition
