# Supplementary material for: Accelerating microbial iron cycling promotes re‐cementation of surface crusts in iron ore regions
Source: Microb Biotechnol. 2020 Aug 19;13(6):1960–71. doi: 10.1111/1751-7915.13646 (PMC7533318; doi:10.1111/1751-7915.13646)
Supplement: Supplementary file 11 — File S1. Supplementary Results. [file MBT2-13-1960-s011.pdf]

File S1. Supplementary Results

Plant growth during canga re-consolidation experiment

Mimosa pudica, *Mimosa camporum*, and *Chloroleucon acaciodes* germinated within the first week in the replicate of treatment D that was provided with seeds (i.e., treatment E), and the plants were in flower by week 12. Spontaneous weeds and grasses grew in the water-only control, presumably germinated from the seed bank that blew into the canga stockpile before construction (this treatment was constructed first and received canga from the surface of the stockpile). By week 24, grasses and weeds had spread to the other treatments, and plant cover increased progressively in all treatments over the 40 weeks of wetting and drying cycles (Figure S3).

Microbial community analysis of canga after drying

Microbial communities detected on the rock material from the uninoculated and inoculated treatment at end harvest (i.e., after 6 months drying) were dominated by Firmicutes (30-45%) in all except the inoculated treatment at 10 cm where Proteobacteria dominated. In contrast, rocks from the water-only control and untreated control were dominated by Actinobacteria or Proteobacteria sequences (Figure S5). Major OTUs in the uninoculated and inoculated treatment were *Clostridium* species that were not abundant in pore waters throughout the experiment (Figure S6). Potential Fe and/or metal cycling lineages were identified in the rocks from the uninoculated and inoculated treatment and included *Geobacter* (up to 2.7%) *Gallionellaceae*, (up to 5.5%) and *Curvibacter* (up to 1.8%, see Figure S6). These had been detected at low abundances (<0.38% of sequences) in pore waters during the experiment and were almost completely absent on rocks from the water-only control and the untreated control (Figure S6). A *Geobacter* (OTU 1932), different to the OTUs present in the uninoculated and

uninoculated treatment, was detected in the water-only control at 0.8% (Supporting File S7). Major OTUs in the untreated control classified within the Actinobacteria and major OTUs in the water-only control included the same Actinobacteria OTUs, as well as a *Thaumarchaeota* (OTU 13) and *Nitrosotalea* (OTU 3) that were detected in pore waters throughout the experiment (Figure S6).
