# Supplementary material for: Accelerating microbial iron cycling promotes re‐cementation of surface crusts in iron ore regions
Source: Microb Biotechnol. 2020 Aug 19;13(6):1960–71. doi: 10.1111/1751-7915.13646 (PMC7533318; doi:10.1111/1751-7915.13646)
Supplement: Supplementary file 13 — File S3. Supplementary Methods. [file MBT2-13-1960-s013.pdf]

File S3. Supplementary Methods

Preparation of microbial consortia

A goethite-reducing enrichment culture (Gagen *et al.*, 2019) was transferred to anaerobically prepared modified Wolfe's mineral medium (MWMM) (Emerson and Merrill Floyd, 2005) with 30 mM glucose and canga as iron source. After two passages on MWMM with canga at 1g/50mL (approx. 3 weeks each) and a third passage on MWMM with canga at 25g/50mL, six well-grown cultures (50 mL each) were inoculated to 16L fresh MWMM medium with goethite (100 g) and incubated for 4 weeks. Subsequently two well-grown cultures (50 mL each) were also inoculated into 9 L fresh MWMM with goethite (250 g) and incubated for one week. These cultures on MWMM medium (16 L and 9 L) were combined for use in the field-scale experiment. The composition of the microbial community on MWMM was determined by amplicon sequencing as per Gagen et al., (2018) and data is available under BioProject number PRJNA634792.

A neutrophilic iron-oxidizing culture was enriched from a small pool of water perched on canga in Serra Sul, Carajás, Brazil (S06°24'01.7" W050°22'23.8") by multiple passages on FeS gradient tubes (with MWMM as the basal medium), as described in Emerson & Floyd (2005). After 10 passages (routinely 10-14 days each growth), the culture was inoculated to 64 mL liquid MWMM medium in two 160 mL serum vials, fed daily with $FeCl_2$ and air as per Emerson & Floyd (2005) for 4 weeks, before each were inoculated into 2.5 L in 5 L bottles and fed daily for 10 days, before use in the field-scale experiment. The composition of the culture was determined at the 4th passage by amplicon sequencing as per Gagen et al. (2018) and is available under BioProject number PRJNA634783.

A phototrophic consortium established from a pooled mixture of samples collected from Serra Sul and Serra Norte, Carajás, Brazil (small pools perched on canga at each of the following locations, S06°24'01.7" W050°22'23.8", S06°23'50.7" W050°21'19.1",

S06°23’50.5” W050°21’27.5”, S06°01’37.9” W050°16’44.9”) was enriched on BG-11 medium (Stanier *et al.*, 1971) on a windowsill in filtered light for 9 months (3 passages) before being adapted to and grown on MWMM medium (8 weeks adaption, then the transferred (2x 2L) culture was grown for 4 weeks before use in the field-scale experiment). Microbial community composition of the phototrophic consortium (an even mixture of two cultures named ‘22K’ and ‘CON’) was determined via whole genome sequencing using an Illumina NextSeq 500, considering a fragment size of 75 bp, paired-end reads. The sequencing library was prepared using the Nextera DNA Library Prep Kit as per manufacturer’s instructions and sequencing was with the NextSeq 500/550 (150 cycle) kit. Data is available under BioProject number PRJNA634792. Composition of the metagenome was determined based on annotations from MG-RAST (Keegan *et al.*, 2016), requiring a minimum sequence alignment match to annotated sequences from the RefSeq database using default parameters: minimum identity cut-off values of 60%, minimum alignment lengths of 15 bp and maximum e-values of 1-e−5.

DNA extraction, amplicon sequencing and sequence processing

DNA was extracted using the DNeasy® Powersoil® DNA isolation kit (Qiagen, Hilden, Germany). For pore water samples, Sterivex™ filter units were cracked open in the laboratory and the filter removed using sterile tweezers, cut into small pieces and placed directly into a screw cap tube for DNA extraction. For liquid samples that were not collected on filters (i.e., some of the difficult-to-filter samples from treatment B), 10 ml was centrifuged in the laboratory for DNA extraction. For end-point rock samples approximately 0.5 g of canga was used for extraction. The V6-V8 region of the extracted DNA was amplified using 926f and 1392r primers and sequenced on a MiSeq Sequencing System (Illumina, San Diego, CA, USA) using paired end sequencing with V3 300 bp chemistry.

Sequencing and analysis using MOTHUR (Schloss *et al.*, 2009) and the Silva reference database SSU Ref NR 99 v132 (Quast *et al.*, 2012; Yilmaz *et al.*, 2013) was as per the MiSEQ SOP (Kozich *et al.*, 2013) with modifications outlined in Gagen (2018). Operational Taxonomic Units (OTUs) that were differentially represented in the water-only control or inoculated or uninoculated treatment were determined using Metastats (Paulson *et al.*, 2011) in MOTHUR. Sequences have been submitted to the National Center for Biotechnology Information (NCBI) Sequence Read Archive under BioProject number PRJNA609832.

Chemical analyses

Porewater samples were treated at 100 °C for 30 minutes in a water bath or heating block before chemical analysis, as part of Australian Biosecurity release procedures. For trace metal analysis, filtered water samples were then acidified to a final concentration of $7\%_{(aq)}$ nitric acid and digested in a MARS Xpress microwave with Teflon tubes (10 min at 160 ºC, followed by 10 min at 170 ºC). The digested samples were diluted to final concentration $5\%_{(aq)}$ nitric acid and analyzed by inductively coupled plasma optical emission spectrophotometry (ICP-OES) using a Perkin Elmer Optima 7300DV with argon as the plasma gas at 15 L min^{-1}. The samples were analyzed for soluble metals (detection limits in ppb given in parentheses): Al (1.2), As (6.3), B (1.5), Ba (0.04), Ca (0.5), Cd (0.1), Co (0.4), Cr (0.4), Cu (0.4), Fe (0.3), K (0.3), Mg (0.1), Mn (0.04), Mo (0.6), Na (0.2), Ni (0.5), P (2.9), Pb (1.7), S (0.2), Se (13) and Zn (0.2).

For volatile fatty acids, a Shimadzu Prominence HPLC system (Kyoto, Japan) was used to determine the presence of formic, lactic, acetic, butyric, propionic, succinic, *iso*valeric and n-valeric acids and glucose in pore waters. Separation and identification of acids from 20 µL sample was achieved using an Agilent Hi-Plex H Column (8 µm, 7.7 x 300 mm, Agilent

Technologies, Australia) at 35 °C using a mobile phase of 14 mM H_2SO_4 at 0.5 ml/min. Limit of detection for all compounds was 2 ppm.

Nitrogen oxides, ammonia and phosphate concentrations were determined using a Lachat QuikChem8500 Series 2 Flow Injection Analyzer, and the software Omnion 3.0. Specifically, nitrite (as NO_2-N, range 0.03-12 ppm) was determined using QuikChem Method 31-107-05-1-A. Briefly, this involves diazotization with sulfanilamide under acidic conditions, before the diazonium ion is coupled with N-(1-naphthyl)ethylenediamine dihydrochloride. The resulting pink product absorbs at 520 nm. Nitrate (as NO_x-N, range 0.03-12 ppm) was determined by QuikChem Method 31-107-04-1-A where nitrate is quantitatively reduced to nitrite by passage of the sample through a copperized cadmium column. The nitrite (reduced nitrate plus original nitrite) is then determined as previously and nitrate concentrations are obtained by subtracting nitrite values (which are simultaneously analyzed) from the nitrite+nitrate values. Phosphate (as PO_4-P, range 0.02 - 8 ppm) was determined by QuikChem Method 31-115-01-1-G. Briefly, ammonium molybdate and antimony potassium tartrate react in an acid medium with phosphate to form an antimony-phospho-molybdate complex. This complex is reduced by ascorbic acid and the resulting blue-coloured complex produced (which absorbs at 880 nm) is proportional to the concentration of orthophosphate in the sample. Ammonia (as NH_3-N, 0.03-12ppm) was determined by QuikChem Method 10-107-06-2-O). When ammonia is heated with salicylate and hypochlorite in an alkaline phosphate buffer, an emerald green colour is produced which is proportional to the ammonia concentration. The colour (which absorbs at 630 nm) is intensified by the addition of sodium nitroprusside.

Scanning electron microscopy

Subsamples of consolidated canga (or unconsolidated canga fragments in the case of the untreated and the water-only control) were dehydrated at 40 °C for a week, resin-embedded (RenLam® M-1 hardened with Ren® HY 956) and made into petrographic sections polished to 0.25 µm. The samples were examined using a JEOL7100 field emission gun scanning electron microscope in backscatter electron (BSE) mode, and energy dispersive X-ray spectroscopy was carried out at an accelerating voltage of 15 kV. Prior to examination, samples were degassed at 50 °C for at least 12 hours and coated with 20 nm carbon using a Quorum Q150T coater.

References

Emerson, D., and Merrill Floyd, M. (2005) Enrichment and isolation of iron-oxidizing bacteria at neutral pH. In *Methods in Enzymology*: Academic Press, pp. 112-123.

Gagen, E.J., Zaugg, J., Tyson, G.W., and Southam, G. (2019) Goethite reduction by a neutrophilic member of the alphaproteobacterial genus *Telmatospirillum*. *Front Microbiol* **10**: 2938.

Gagen, E.J., Levett, A., Shuster, J., Fortin, D., Vasconcelos, P.M., and Southam, G. (2018) Microbial Diversity in Actively Forming Iron Oxides from Weathered Banded Iron Formation Systems. *Microbes Environ* **33**: 385-393.

Keegan, K.P., Glass, E.M., and Meyer, F. (2016) MG-RAST, a Metagenomics Service for Analysis of Microbial Community Structure and Function. In *Microbial Environmental Genomics (MEG)*. Martin, F., and Uroz, S. (eds). New York, NY: Springer New York, pp. 207-233.

Kozich, J.J., Westcott, S.L., Baxter, N.T., Highlander, S.K., and Schloss, P.D. (2013) Development of a dual-index sequencing strategy and curation pipeline for analyzing amplicon sequence data on the MiSeq Illumina sequencing platform. *Appl Environ Microbiol* **79**: 5112-5120.

Paulson, J.N., Pop, M., and Bravo, H.C. (2011) Metastats: an improved statistical method for analysis of metagenomic data. *Genome Biol* **12**: P17.

Quast, C., Pruesse, E., Yilmaz, P., Gerken, J., Schweer, T., Yarza, P. et al. (2012) The SILVA ribosomal RNA gene database project: improved data processing and web-based tools. *Nucl Acids Res* **41**: D590-D596.

Schloss, P.D., Westcott, S.L., Ryabin, T., Hall, J.R., Hartmann, M., Hollister, E.B. et al. (2009) Introducing mothur: open-source, platform-independent, community-supported software for describing and comparing microbial communities. *Appl Environ Microbiol* **75**: 7537-7541.

Stanier, R.Y., Kunisawa, R., Mandel, M., and Cohen-Bazire, G. (1971) Purification and properties of unicellular blue-green algae (order Chroococcales). *Bacteriol Rev* **35**: 171-205.

Yilmaz, P., Parfrey, L.W., Yarza, P., Gerken, J., Pruesse, E., Quast, C. et al. (2013) The SILVA and "All-species Living Tree Project (LTP)" taxonomic frameworks. *Nucl Acids Res* **42**: D643-D648.
